# Proteomic Characterization of Two Medically Important Malaysian Snake Venoms, *Calloselasma rhodostoma* (Malayan Pit Viper) and *Ophiophagus hannah* (King Cobra)

**DOI:** 10.3390/toxins10110434

**Published:** 2018-10-26

**Authors:** Sugita Kunalan, Iekhsan Othman, Sharifah Syed Hassan, Wayne C. Hodgson

**Affiliations:** 1 Jeffrey Cheah School of Medicine and Health Sciences, Monash University Malaysia, Jalan Lagoon Selatan, Bandar Sunway, Selangor Darul Ehsan 47500, Malaysia; sugitakunalan@hotmail.com (S.K.); iekhsan.othman@monash.edu (I.O.); sharifah.syedhassan@monash.edu (S.S.H.); 2 Monash Venom Group, Department of Pharmacology, Faculty of Medicine, Nursing and Health Sciences, Monash University, Victoria 3800, Australia

**Keywords:** *Calloselasma rhodostoma*, *Ophiophagus hannah*, toxin, proteomics, venomics, venom proteome

## Abstract

*Calloselasma rhodostoma* (CR) and *Ophiophagus hannah* (OH) are two medically important snakes found in Malaysia. While some studies have described the biological properties of these venoms, feeding and environmental conditions also influence the concentration and distribution of snake venom toxins, resulting in variations in venom composition. Therefore, a combined proteomic approach using shotgun and gel filtration chromatography, analyzed by tandem mass spectrometry, was used to examine the composition of venoms from these Malaysian snakes. The analysis revealed 114 proteins (15 toxin families) and 176 proteins (20 toxin families) in Malaysian *Calloselasma rhodostoma* and *Ophiophagus hannah* species, respectively. Flavin monoamine oxidase, phospholipase A_2_, phosphodiesterase, snake venom metalloproteinase, and serine protease toxin families were identified in both venoms. Aminopeptidase, glutaminyl-peptide cyclotransferase along with ankyrin repeats were identified for the first time in CR venom, and insulin, c-type lectins/snaclecs, hepatocyte growth factor, and macrophage colony-stimulating factor together with tumor necrosis factor were identified in OH venom for the first time. Our combined proteomic approach has identified a comprehensive arsenal of toxins in CR and OH venoms. These data may be utilized for improved antivenom production, understanding pathological effects of envenoming, and the discovery of biologically active peptides with medical and/or biotechnological value.

## 1. Introduction

Approximately 5.5 million people are victims of snake bites every year, causing about 400,000 amputations, and 20,000 to 125,000 fatalities [1], representing a global public health burden. Despite its destructive propensity, snake venom has been studied to identify lead compounds for use as therapeutic agents. Promising reports of antimicrobial (bacteria, virus, fungi, and protozoa), anti-inflammatory, antinociceptive, anti-cancer, anti-hypertensive, anti-coagulation, and anti-fibrinolytic activities of snake venoms [2,3,4,5,6,7] have initiated more intensive investigations of venom and venom glands using venomics [8,9]. However, it is widely hypothesized that feeding and environmental conditions influence the concentration and distribution of snake venom components, causing variation between individual, species, genus, and families [10]. Such variation can have major medical implications for the treatment of human snakebite victims. Thus, the reason for the conception of venomics and optimism is that characterization of the large protein variability within snake venoms may contribute to a deeper understanding of their biological as well as pathological effects.

In this study, we investigated the proteomic composition of *Calloselasma rhodostoma* and *Ophiohagus hannah* venoms, two medically important snakes found in Malaysia [11]. Both venoms are a rich source of proteins that have been reported to contain biologically active peptides with medical and biotechnological value. One such example is Ancrod (or Viprinex^®^), a snake venom serine protease purified from *C. rhodostoma* venom. Ancrod is a thrombin-like enzyme that breaks down fibrinogen and was developed for the treatment of ischaemic stroke and myocardial infarction as well as deep-vein thrombosis [12,13]. However, Ancrod has been suspended from use due to inconclusive clinical trial results [14,15]. Another example is the α-neurotoxin, hannalgesin, that was derived from a small peptide called prohanin, originating from the venom of *O. hannah* [16]. Despite being an α-neurotoxin, hannalgesin does not paralyze skeletal muscles, but provides analgesic effects. However, the development of the drug was terminated in the preclinical phase [17,18].

Until recently, there have been relatively few reports on the proteomic profile of these two medically important Malaysian snakes. In a comprehensive study by Tang et al., a total of 96 proteins were identified for *C. rhodostoma* venom [19]. The study employed a series of chromatographic separations, followed by in-gel tryptic digestion of one-dimension (1D) gel electrophoresis proteins and, lastly, identification based on mass spectrometry. In earlier investigations, *C. rhodostoma* venom proteins were studied using two-dimension (2D) gel electrophoresis together with mass spectrometry. Ali et al. (2013) reported the presence of disintegrin, kallikrein (thrombin-like protein), L-amino acid oxidase (LAAO), lectin, and phospholipase A_2_ (acidic, basic, and neutral) as well as snake venom metalloproteinases (SVMPs) belonging to six protein families, whereas Vejayan et al., (2014) presented evidence for 26 proteins that could be grouped into six protein families, as demonstrated by Ali et al. [20,21]. Despite the positive identification of toxins, there were 89 unidentified spots that indicates a substantial gap in the complete venom profiling of the *C. rhodostoma* venom. Apart from the relatively small number of identified proteins, both studies did not provide information on the relative abundance of the identified proteins.

An elaborate venom-gland transcriptomic and venom proteomic study of *O. hannah* venom revealed 128 transcripts and 45 proteins, which were classified into 16 individual protein families [22]. The major protein families reported were three-finger toxin (3FTx), SVMP, cysteine-rich secretory protein (CRiSP), LAAO, Vespryn, phospholipase A_2_ (PLA_2_), and cobra venom factor (CVF). In addition, nine low abundance (≤2%) protein families were detected. The results of the study were compared with the Balinese (Indonesian) *O. hannah* venom investigation by Vonk et al. (2013) [23]. In contrast, they reported 73 proteins using a combination of shotgun and 2D gel electrophoresis. The major protein families described for Balinese *O. hannah* venom were highly similar to the Malaysian *O. hannah* venom, in which the most abundant toxins were 3FTxs and SVMPs. Although similarities were detected, we know that intraspecies toxin composition variations exist between venoms. As mentioned above, Vejayan et al. (2014) also investigated the proteomic profile of Malaysian *O. hannah* venom together with *C. rhodostoma* venom. Only 15 proteins belonging to five toxin families could be deduced from 50 spots that were isolated from the 2D gel. The large number of uncharacterized proteins, coupled with the discrepancy of the number of identified proteins between the Malaysian and Indonesian species, indicates the need for further proteomic exploration of Malaysian *O. hannah* venom.

In this work, we employed a combination of two proteomic approaches to provide a more comprehensive proteomic profile of two medically significant venomous snakes from Malaysia. The first approach involves a direct tryptic digest of freeze-dried crude venom proteins followed by ESI-LC-MS/MS, whereas the second approach subjects the crude venom proteins to a molecular-weight based separation prior to tryptic digestion and subsequent ESI-LC-MS/MS. Using this strategy, we report a total of 114 proteins for *C. rhodostoma* and 176 proteins for *O. hannah* venoms, successfully identifying more proteins and peptides than previously reported.

## 2. Results

We characterized the venom composition of two Malaysian snakes, i.e., *Calloselasma rhodostoma* and *Ophiophagus hannah*, using two proteomic approaches: Direct tryptic digest of freeze-dried crude venom proteins followed by liquid chromatography coupled with electrospray tandem mass spectrometry (shotgun-ESI-LC-MS/MS) and tryptic digest of gel-filtration chromatography venom protein fractions and subsequent liquid chromatography coupled with electrospray tandem mass spectrometry (GF-ESI-LC-MS/MS). The combined approaches revealed a diverse set of data that has been summarized in Table 1, Table 2, Table 3 and Table 4 and is discussed in detail below. A complementary visual representation of the crude venom composition is also presented.

### 2.1. SDS-PAGE of Crude Venom Proteins

**Figure 1:**
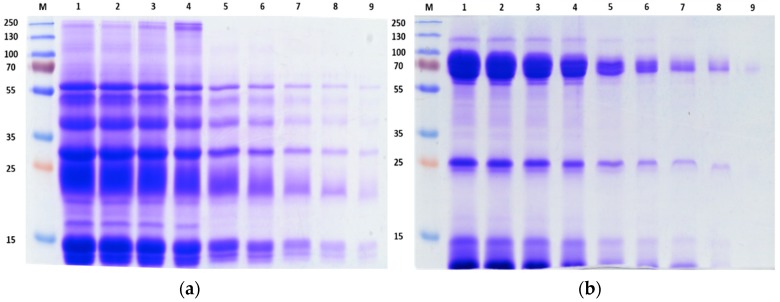
One-dimension electrophoretic profile of (**a**) *C. rhodostoma* and (**b**) *O. hannah* venoms. 12% SDS-PAGE gel picture showing the individual venoms prepared and run in various concentrations. M represents the molecular weight standard in kDa. The lanes (1–9) contain 100, 80, 60, 40, 20, 10, 5, 2.5, and 1 μg of venom proteins, respectively.

The overall protein composition of *C. rhodostoma* and *O. hannah* venoms was assessed by 1D SDS-PAGE (Figure 1). The Coomassie Brilliant blue staining showed varying electrophoretic (molecular weight-distribution) protein profiles. This is not surprising as the venom proteins belong to different snake families, one a Viperidae (*C. rhodostoma*) and the other an Elapidae (*O. hannah*). The separations revealed that both venoms were composed of highly heterogeneous proteins varying in terms of band intensity as well as migration. Although staining was observed in the entirety of the gel background for both venoms, *O. hannah* venom appears to have more large molecular weight proteins compared to *C. rhodostoma* venom. *C. rhodostoma* venom proteins were generally <60 kDa whereas *O. hannah* venom proteins appeared to be concentrated in several regions, namely 70 kDa, 25 kDa, and 10 kDa.

The crude venom protein patterns observed in both gels show strong similarity with electrophoretic profiles from previous studies using Malaysian species [22,24]. A visual representation of the crude venom protein pattern provides an indication of possible geographic and species variation as well as illustrates the complexity of the sample being investigated.

### 2.2. Gel Filtration Separation of Crude Snake Venom Using Superdex G-75

**Figure 2:**
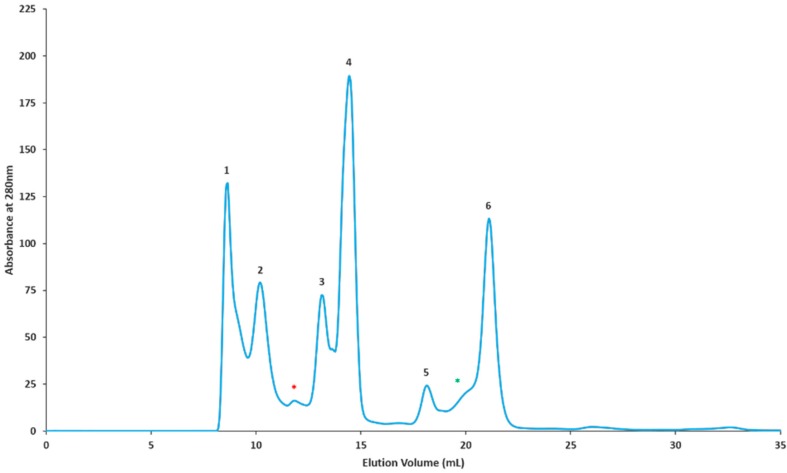
Superdex G75 chromatographic profile of Malaysian *C. rhodostoma* venom. Venom (10 mg) was dissolved in 10 mM ammonium acetate (250 μL), pH 7.0 and loaded onto a pre-equilibrated column. Pre-equilibration and isocratic elution was carried out using the sample buffer. The chromatographic separation yielded six prominent peaks (1–6) and two minor fractions (marked *). Individual runs were repeated (at least 10 times). Fractions were pooled, desalted, concentrated, and subjected to in-solution tryptic digestion before analysis by ESI-LC-MS/MS.

**Figure 3:**
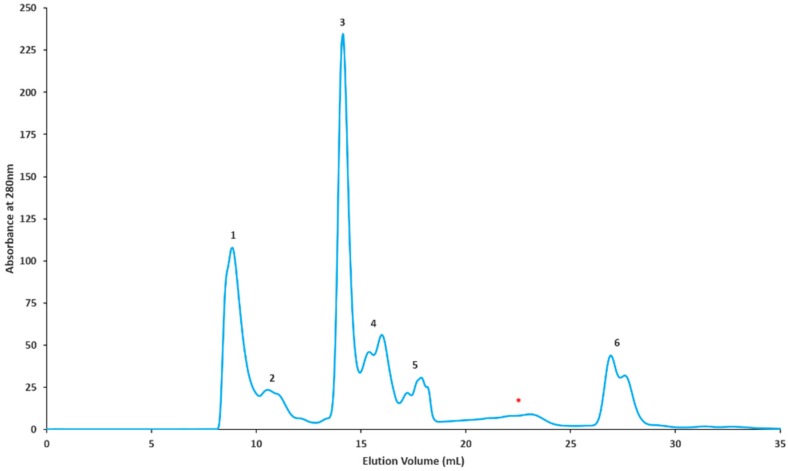
Superdex G75 chromatographic profile of Malaysian *O. hannah* venom. Venom (10 mg) was dissolved in 10 mM ammonium acetate (250 μL), pH 7.0 and loaded onto a pre-equilibrated column. Pre-equilibration and isocratic elution was carried out using the sample buffer. The chromatographic separation yielded six prominent peaks (1–6) and one minor fraction (marked *). Individual runs were repeated (at least 10 times). Fractions were pooled, desalted, concentrated, and subjected to in-solution tryptic digestion before analysis by ESI-LC-MS/MS.

For the GF-ESI-LC-MS/MS based approach, *C. rhodostoma* and *O. hannah* venoms were subjected to a molecular weight separation according to Section 4.2.4. The proteins were eluted based on their molecular sizes, beginning with large molecular weight proteins. *C. rhodostoma* separation yielded six distinctive peaks and two minor fractions, i.e., a total of eight fractions (Figure 2). Similarly, *O. hannah* venom separation yielded six peaks and one minor fraction, with a total of seven fractions (Figure 3). All resulting fractions were pooled according to their individual peaks and subjected to in-solution tryptic digestion prior to characterization.

### 2.3. Snake Venom Proteomic Characterization Using Shotgun Approach (Shotgun-ESI-LC-MS/MS)

Using the shotgun-ESI-LC-MS/MS approach, a total of 47 proteins belonging to 12 snake venom protein families were identified in *C. rhodostoma* venom (Table 1). The major protein families categorized were c-type lectin/snaclec (CTL/snaclec), phosphodiesterase (PDE), PLA_2_, SVMP, 5′-nucleotidase (5′-NTD), and snake venom serine protease (SVSP), followed by minor flavin monoamine oxidase (FMO), phospholipase B (PLB), CRiSP, glutaminyl-peptide cyclotransferase (QPCT), Trysinogen, and Ankyrin protein families. The latter three proteins were solely identified using this approach. For *O. hannah* venom, 76 proteins were identified from 19 toxin families. Quantitatively, there were two prominent families that made up the majority of the total venom, 3FTx with 23 peptides, followed by SVMP with 21 peptides (Table 2). The minority of venom was made up of Kunitz-type, CRiSP, FMO, PDE, 5′-NTD, PLA_2_, nerve-growth factor (NGF), acetylcholinesterase (AChE), complement C3 homolog (CC3H), endonuclease, neprilysin, ohanin/vespryn, platelet-derived growth factor/vascular endothelial growth factor (PDGF/VEGF), and SVSP protein families. Some notable proteins identified included the presence of insulin growth factors, hepatocyte growth factor activator, and CTL/snaclec peptides in *O. hannah* venom, which will be discussed in detail below. 5′-NTD and neprilysin proteins were exclusively detected using this approach. 

### 2.4. Snake Venom Proteomic Characterization Using Gel Filtration Protein Fractions (GF-ESI-LC-MS/MS)

All resulting peptides from digested individual chromatographic fractions were classified according to protein families as per the previous section. From eight chromatographic fractions obtained for *C. rhodostoma* venom, a total of 99 proteins belonging to 12 protein families were detected (Figure 2, Table 3). The *C. rhodostoma* protein families were predominantly comprised of SVMPs as well as SVSPs and PDEs, while others families such as FMO, CTL/snaclec, PLA_2_, PLB, NGF, 5′-NTD, endonuclease, and CRiSP, were represented by lesser numbers of peptides. Of interest, the aminopeptidase protein family was newly discovered in *C. rhodostoma* venom using this proteomic approach. Other proteins not identified using the shotgun technique included endonucleases and NGFs (Table 3). For *O. hannah* venom, 150 proteins from 18 major families were observed for the seven chromatographic fractions (Figure 3, Table 4). The OH venom families consisted of 3FTx, SVMP, PLA_2_, CRiSP, PDE, Complement C3, Kunitz-type, Insulin, SVGF, SVSP, ohanin/vespryn, FMO, endonuclease, CTL/snaclec, AChE, NGF, PDGF/VEGF, and PLB. Interestingly, the presence of the single PLB peptide in the venom was detected only using this proteomic approach.

### 2.5. Summary of Identified C. rhodostoma and O. hannah Snake Venom Proteins

**Figure 4:**
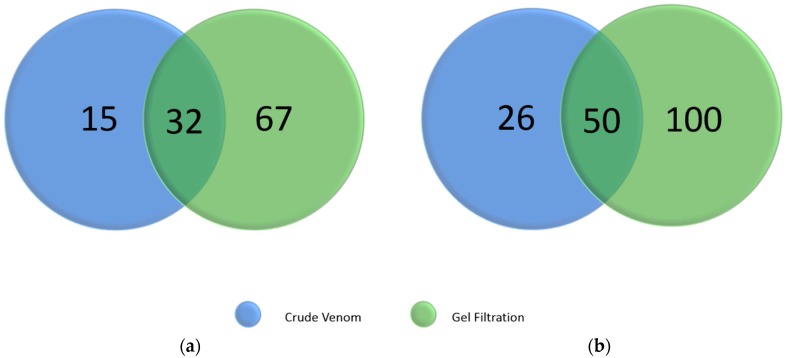
A two-way Venn diagram depicting the overlap of the number of proteins identified by two proteomic strategies for (**a**) *C. rhodostoma* and (**b**) *O. hannah* venoms. The strategies employed crude venom and gel filtration fractions to comprehensively characterize the entire venom proteome. The proteomic analyses were performed via a combination of nanoflow liquid chromatography coupled with electrospray tandem mass spectrometry (ESI-LC-MS/MS) and automated de novo sequencing. 32 and 50 proteins were common between the two data sets for *C. rhodostoma* and *O. hannah*, respectively. Both venoms demonstrated a higher number of proteins identified in the gel filtration strategy compared to the crude venom strategy. The identified proteins were matched using the UniProt database (Serpentes) and subsequently categorized according to individual toxin family.

**Figure 5:**
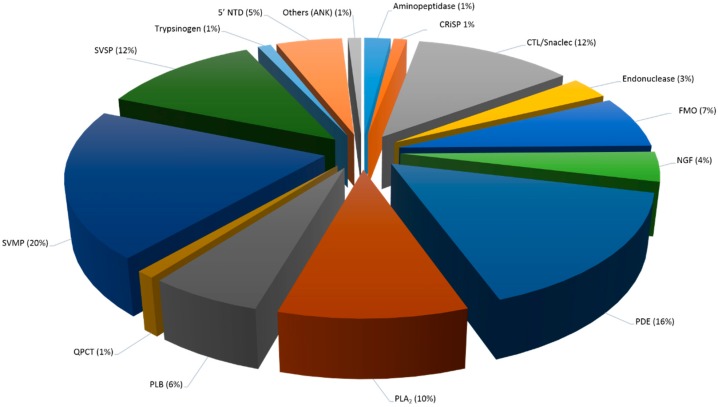
The proteome of Malaysian *Calloselasma rhodostoma* venom. Pie chart represents the relative abundance (expressed in percentage) of protein families identified in the whole crude venom. A total of 15 toxin families were determined by a combination of nanoflow liquid chromatography coupled with electrospray tandem mass spectrometry (ESI-LC-MS/MS) and automated de novo sequencing. Abbreviations: CRiSP: Cysteine-rich secretory proteins; CTL: C-type lectin; FMO: Flavin monoamine oxidase; NGF: Nerve-growth factor; PDE: Phosphodiesterase; PLA_2_: Phospholipase A_2_; PLB: Phospholipase B; QPCT: Glutaminyl-peptide cyclotransferase; SVMP: Snake venom metalloproteinase; SVSP: Snake venom serine proteinase; ANK: Ankyrin repeats.

**Figure 6:**
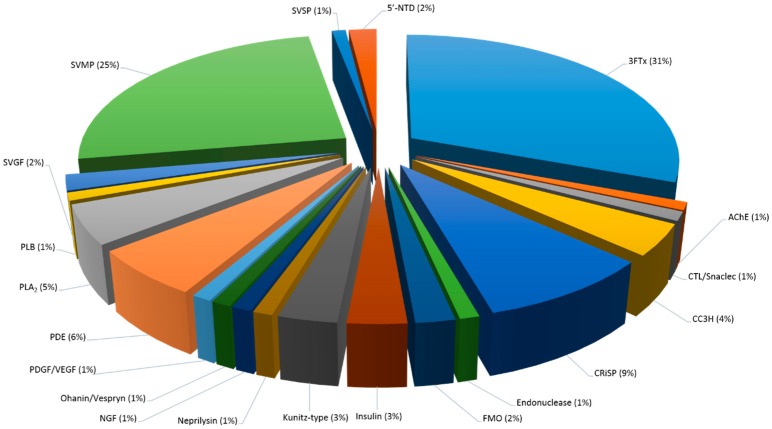
The proteome of Malaysian *Ophiophagus hannah* venom. Pie chart represents the relative abundance (expressed in percentage) of protein families identified in the whole crude venom. A total of 20 toxin families were determined by a combination of nanoflow liquid chromatography coupled with electrospray tandem mass spectrometry (ESI-LC-MS/MS) and automated de novo sequencing. Abbreviations: 3FTx: Three-finger toxins, AChE: Acetylcholinesterase; CTL: C-type lectin; CRiSP: Cysteine-rich secretory proteins; FMO: Flavin monoamine oxidase; NGF: Nerve-growth factor; PDGF/VEGF: Platelet-derived growth factor/vascular endothelial growth factor; PDE: Phosphodiesterase; PLA_2_: Phospholipase A_2_; PLB: Phospholipase B; QPCT: Glutaminyl-peptide cyclotransferase; SVGF: Snake venom-growth factor; SVMP: Snake venom metalloproteinase; SVSP: Snake venom serine protease.

To obtain an overview of the major protein classes in Malaysian *C. rhodostoma* and *O. hannah* snake venoms, we combined two proteomic approaches that have made it possible to detect a higher number of proteins than previously reported. Our proteomic analysis revealed 114 proteins, belonging to 15 major protein families, for *C. rhodostoma* venom, and 176 proteins, belonging to 20 protein families, for *O. hannah* venom (Figure 4, Figure 5 and Figure 6, Table 5). Amongst these identified protein families are several new, previously undiscovered proteins for these venoms. These include aminopeptidase, glutaminyl-peptide cyclotransferase, and Ankyrin repeat for *C. rhodostoma* venom and insulin, CTL/snaclec, and SVGF (hepatocyte growth factor activator, macrophage colony stimulating factor, tumor necrosis factor receptor) for *O. hannah* venom (Table 5).

Using the shotgun approach, a total of 47 and 76 proteins were detected in CR and OH venoms, whereby 15 and 26 proteins were exclusively identified using the shotgun approach. In comparison, 99 and 150 proteins were identified using the gel-filtration approach, with 67 and 100 proteins being detected exclusively with this approach (Figure 4). Although the most common and widely used liquid chromatography separation for venom proteome profiling is reverse-phase C18 HPLC, we have utilized a molecular weight based separation to try to overcome some potential difficulties in the elution of low abundant large molecular weight proteins [25,26]. It appears that prior protein separation, together with the use of the unconventional molecular weight based separation, does indeed allow for decomplexation of crude venom proteins that has enabled a higher number of proteins to be detected.

We were also interested in the identification of low abundant proteins that could be of biological interest as a potential source of active compounds or play an important role in snake envenoming. Thus, the shotgun approach was employed to target potentially indiscernible small molecular weight proteins, even by SDS-PAGE [27]. Looking at the migration and strong intensity of the protein bands in the lower molecular weight range (Figure 1), we believe that the shotgun approach provided the necessary complementary analysis. Thus, combined, the results of both techniques have facilitated a detailed proteomic view of the entire venom components (Table 5, Figure 5 and Figure 6).

#### 2.5.1. Snake Venom Protein Classes in Both *C. rhodostoma* and *O. hannah* Venoms

Generally, there are some protein families that are commonly present in the venoms of all snake families, i.e., Viperidae, Atractaspididae; Elapidae, and Colubridae, while others are restricted to certain families [28]. For instance, viper venoms usually induce hemorrhagic, hypotensive, and inflammatory effects due to high concentrations of metalloproteases, serine proteases, and C-type lectins, whereas elapid venoms usually induce neurotoxic symptoms owing to large amounts of pre- or post-synaptic neurotoxins. While these venoms may display different venom presentations, we identified a total of 11 families that were shared between these two snake families (Table 5, Figure 5 and Figure 6). The toxin families between the two snakes are compared and discussed below.

##### SVMP

This class of proteins represents a major toxin family in viper venoms, including Vipernaes and Crotalinaes. In Elapidaes, the proteins make up smaller proportions of the venom. The snake venom metalloproteinases (SVMPs) are known to display a wide range of physiological activates, such as hemorrhagic, fibrinolytic, prothrombin activating, blood coagulation factor X activating, apoptotic, platelet aggregation inhibition, proinflammatory, and inactivation of blood serine proteinase inhibitors [29]. Based on their molecular weight differences and domain structure, the SVMPs are divided into three main classes: P-I, P-II, and P-III. Comparatively, P-I SVMPs are relatively small at 20–30 kDa and contain a single catalytic metalloproteinase (MP) domain. P-II SVMPs, with molecular weight between 30–60 kDa, consist of an MP domain and an additional disintegrin domain. P-III proteins are the largest at 60–100 kDa, composed of both the MP and disintegrin domains as well as a cysteine-rich domain. In general, the P-IIIs are considered the most potent of SVMPs, followed by the P-IIs and the least hemorrhagic P-Is. Interestingly, all classes of SVMPs are responsible for the classic Viperid envenoming effect, which is the ability to cause hemorrhage at the bite site [30,31]. Paradoxically, novel human therapeutics may be achieved from the bioactive peptides of SVMPs because of their haemorrhage, coagulopathy, and inflammatory responses. Several examples of SVMPs with clinical utility include Alfimeprase (*A. contortrix contortrix*), Integrelin (*S. miliarius barbouri*), Aggrastat (*E. carinatus*), and NN-PF3 (*N. naja*) [6]. In this study, we report 20% SVMPs for the venom of the viperid *C. rhodostoma* and 25% for the venom of the elapid *O. hannah* (Table 5, Figure 5 and Figure 6). Although the results appear contrasting, previous studies have shown significant amount of SVMPS in *O. hannah* with 12% (Balinese specimen) and 24% (Malaysian specimen) [22,23]. However, it is important to note that despite the high amounts of SVMP detected, it is not the primary toxin family identified in the Elapidae venom. In contrast, even though only 20% were detected in *C. rhodostoma* venom, this was the largest toxin family represented in this Viperidae venom.

##### SVSP

Our study revealed a distinct difference in the abundance of snake venom serine proteinases (SVSPs) identified in *C. rhodostoma* venom (12%) compared to *O. hannah* venom (1%) (Table 5, Figure 5 and Figure 6). This is not unexpected, as SVSPs are commonly found in viperid venoms, but only in relatively low amounts in some Elapid venoms [32]. Together with SVMPs, the SVSPs are proteolytic enzymes that play important roles during snake envenoming. This family of proteins is known to exert hemostatic effects on prey by acting on various components of the coagulation cascade, on the fibrinolytic and kallikrein–kinin systems and cells to produce an imbalance of their systems [33,34]. The SVSPs that have been biologically characterized have both, or either, fibrinogenolytic and fibrinolytic activities and based on that, they either promote or inhibit coagulation. Some SVSPs are also known as ‘thrombin-like’ enzymes or abbreviated to SVTLE (snake venom thrombin like enzymes) due to their ability to mimic the fibrinogenolytic activities of thrombin [35]. Thrombin-like enzymes, ancrod and ancrod-2, were discovered in *C. rhodostoma* venom in high abundance using both shotgun and gel-filtration methods. First purified in 1967, ancrod was described as a potential anticoagulant and thoroughly researched for many years due to its potential clinical utility [36]. In *O. hannah* venom, only two SVSPs were identified, Alpha and beta fibrinogenase OhS1 and Neuroendocrine convertase 1. The Alpha and beta fibrinogenase OhS1 was also identified by Tan (2015) in the Malaysian king cobra species and is purported to possess potent fibrinogenolytic and amidolytic activities without any haemorrhagic consequences. In venomous animals, the neuroendocrine convertase 1 protein has only been identified in spider and gila monster venoms and the transcript of cone snail venom-glands [37,38,39,40]. The function of this protein is to convert inactive proteins into bioactive components by cleaving after Lys-Arg or after a tetrabasic sequence (Arg-X-Arg/Lys-Arg) [41,42]. Findings postulate that ‘venom proprotein convertase’ is most likely involved in the activation of exendins in gila monster, protoxins in spider venom, and conotoxins in cone snails (cleavage after dibasic residues) [38,43]. It is suggested that proforms of peptide toxins are activated by proteolytic cleavage by venom proprotein convertase, such as neuroendocrine 1 [37].

##### CRiSP

Cysteine-rich secretory proteins (CRiSPs) are relatively small glycoproteins (molecular weight 20 to 30 kDa) with a high degree of amino acid sequence similarity and highly conserved specific pattern of 16 cysteine residues [44,45,46]. This family of proteins are widely distributed and have been established in mammals, *Drosophila*, *Xenopus*, *Heloderma horridum horridum*, *Caenorhabditis elegans*, and *Conus textile*. Some of their proposed biological functions are gamete fusion, sperm maturation, anti-microbial and matrix-degradation, sperm chemoattractant, Ca^2+^ and K^+^ channel blockers, longevity and stress resistance regulators, and protease-like activity [44]. In snakes, they are found in most families, including Viperidae, Elipidae, and Colubridaes, demonstrating Ca^2+^ channel blocker-like properties and cyclic nucleotide-gated channel blocker activities [47,48]. This study revealed 15 CRiSP proteins in *O. hannah* venom (9%), but only one CRiSP protein in *C. rhodostoma* venom (1%) (Table 5, Figure 5 and Figure 6). This finding coincides with previous studies, whereby individual acidic and basic fragments of the same protein were identified. The 15 CRiSPs in *O. hannah* venom showed sequence similarities to 12 different species (Table 2 and Table 4). The ophanin from *O. hannah* venom was first purified and characterized in 2003 and compared to nine other snake venom CRiSP proteins. Based on the phylogenetic tree constructed in that study, it was suggested that *O. hannah* is more closely related to the Viperidae snakes rather than Elapidae snakes owing to the similarity of the nucleotide sequence of ophanin with the Viperidae snakes [49].

##### Phospholipase A_2_

Phospholipase A_2_’s activates the hydrolysis of glycerophospholipids at the sn-2 position of the glycerol backbone to dissociate fatty acids and corresponding 1-acyl lysophospholipids [2,50]. Over the years, the toxic and pharmacological effects presented by these enzymes have been well-documented. Effects include neurotoxic, myotoxic, cardiotoxic, cytotoxic, hemolytic, hypotensive, platelet aggregation, anticoagulant, pro-inflammatory, edematogenic, and bactericidal activities [50,51]. Widespread in nature, PLA_2_ has been identified in venoms from all snake families; i.e., Colubridae, Elapidae, Viperidae, and Hydrophilae [32]. Here, we report relatively high amounts of PLA_2_ enzymes in both *C. rhodostoma* and *O. hannah* venoms with 10% and 5%, respectively (Table 5, Figure 5 and Figure 6). The number of identified PLA_2_ peptides in this study is noticeably higher than reported in previous studies [19,20,21,22,52,53]. Ali et al. (2013), Vejayan et al. (2014), and Tsai et al. (2001) reported the presence of both acidic and basic isoforms of PLA_2_ in *C. rhodotoma* venom, whereas Tang et al. (2016) primarily identified the basic form of the enzyme. Additionally, Tang et al. identified two PLA_2_ peptides in minute amounts with sequence similarity to *N. kaouthia* venom, one of which was an acidic fraction found in less than 0.01% abundance. In our study, we identified a total of 11 PLA_2_ peptides (both acidic and basic isoforms), discovering more than two times the number of PLA_2_ compared to the latest proteomic analysis [19]. PLA_2_ peptides from *C. rhodostoma* venom had similar sequences to PLA_2_ peptides from *O. hannah*, *T. stejnegeri*, *C. atrox*, *T. sabahi*, and *C. horridus* venoms. Tsai et al. and Tan et al. both reported that the major PLA_2_ isolated from the Malaysian king cobra is the acidic isoform of the enzyme [22,52]. Based on the protein score obtained in our study, we are in agreement with their findings. The additional PLA_2_ peptides from *O. hannah* venom showed similarity to sequences from *C. rhodostoma*, *B. fasciatus*, *M. fulvius*, and *T. biscutatus* venoms. However, the higher number of PLA_2_s identified for both venoms here provides evidence that intragenic variations may indeed be influenced by ontogenic factors as suggested by Tan et al. (2015), which in turn will have significant impacts on the outcome of snake envenoming in Malaysia.

##### PLB

Phospholipase type B (PLB) is another member of the phospholipase superfamily. However, compared to PLA_2_, information on this enzyme is relatively scarce. The lack of data could be due to the limited identification of this enzyme in snake venomics analyses and/or the minute amounts of the enzyme that have been detected in snake venoms [54]. PLB hydrolyzes monoacyl phosphatides by liberating free fatty acid and forming glycerophosphoryl derivatives. To date, phospholipase B activity has been reported in snakes by Doery and Pearson (1964), Van Deenen and Haas (1963), Mohamed (1969), Shiloah (1973), and Bernheimer (1987) [55,56,57,58,59]. One study presented hemolytic and cytotoxic activities in *Pseudechis colletti* venom, indicating possible biological effects of this enzyme. In mice, PLB causes myoglobinuria, a muscle destruction occurrence also exerted by PLA_2_. Interestingly, Shiloah (1973) also observed that purified *N. naja* PLA_2_ isoforms presented PLB activity at specific experimental conditions. This finding led to the suggestion that PLB activity found in snake venoms may be due to a dual action by PLA_2_ [5]. In this study, we discovered 6% of PLB peptides in *C. rhodostoma* venom and 1% in *O. hannah* venom (Table 5, Figure 5 and Figure 6). A previous proteome study for the Malaysian *C. rhodostoma* venom reported less than 1% of this enzyme, whereas the Malaysian *O. hannah* venom presented only one PLB peptide, which is in accord with our study. Although, PLB identification and quantification has been limited thus far, Tan (2015) reported that the enzyme is present in some vipers and pit vipers [54,60,61,62]. In a recent proteomic study by Zainal Abidin et al. (2017) this finding was confirmed with a total of 8% and 2% of PLB enzymes reported for Malaysian *T. wagleri* and *C. purpureomaculatus* vipers, respectively. Although the exact pathophysiological role of PLB in venom has not been elucidated, the recent increasing abundance discovered in viper venoms may suggest a more significant role in snake envenoming than first predicted.

##### CTL/snaclec

Classically, CTLs bind to calcium and sugar residues (hence, “C” calcium and lectin). However, the C-type lectin-like proteins of snake venom do not contain the calcium and sugar binding loop and, thus, do not have lectin activity [63,64]. To avoid confusion between classic C-type lectins and C-type lectin-like proteins, the latter was termed snaclec (snake venom c-type lectins). Snaclecs exhibit numerous biological activities, including anticoagulation, pro-coagulation, and platelet modulating (agonist and antagonist) activities [63]. The phenomenon of contrasting platelet activity in snake venom has been reported in several crotalids, such as *H. hypnale*, *D. acutus*, and *C. rhodostoma* species [19,65,66]. Nevertheless, CTLs and snaclecs are considered to be abundant components of snake venom, particularly in vipers. In elapids, only minor amounts (<2%) have been reported in about one third of the family. Here, we report the abundance of CTL/snaclecs in *C. rhodostoma* with 12% compared to 1% in *O. hannah* venom (Table 5, Figure 5 and Figure 6). In *C. rhodostoma* venom, we identified all four subunits of rhodocetin, the platelet aggregation inhibitor snaclec. We also identified both alpha and beta subunits of rhodocytin, the platelet aggregation inducer snaclec that was previously known as aggretin and rhodoaggretin. Previous studies have shown evidence of these proteins grouped as one of the major components in *C. rhodostoma* venom [19,21]. In contrast, Tan et al. (2015) reported that although CTL venom-gland transcripts from the Malaysian and Balinese *O. hannah* venom were discovered, the transcribed genes were not translated into proteins. In this study, we present the first report of snaclec proteins found in *O. hannah* venom. The two CTL/snaclec peptides discovered in *O. hannah* venom showed sequence similarity to the alpha and beta subunits of the platelet aggregation inhibitor, rhodocetin, from *C. rhodostoma* venom.

##### FMO (l-amino Acid Oxidase)

L-amino acid oxidases (LAAOs) belong to the flavin monoamine oxidase (FMO) family and are considered one of the best studied protein families from snake venom. Since the discovery of snake venom LAAOs, many reports have been published on various species of the enzyme. Generally known as flavoenzymes, LAAO catalyzes the stereospecific oxidative deamination of an l-amino acid substrate to produce an α-ketoacid equivalent as well as ammonia and hydrogen peroxide [67,68,69]. Although the pathophysiological role in snake venom has not been defined, researchers have taken advantage of this well-characterized enzyme to study the biological and pharmacological activities of LAAOs. Some of the reported biological effects include hemorrhage, edema, coagulation, and platelet aggregation, whereas pharmacological effects include antimicrobial (virus, bacteria, and parasite) and anti-cancer activities [70]. In a recent snake venom database review, it was reported that 91% of all crotalinae species contained LAAO enzymes and the maximum amount identified thus far is from the crotalinae *R. cotiara*, constituting 20% of the venom. Although important in elapidaes and viperinaes, the enzymes only make up approximately 6% of their venoms [32]. In the current study, we report 7% LAAOs in *C. rhodostoma* venom and 2% in *O. hannah* venom (Table 5, Figure 5 and Figure 6). Although, the relative abundance reported here seems to be much lesser than reported by Ponnudurai et al. (1994), this value is in agreement with Tang et al. (2016), who reported a little less than 7% of LAAO in *C. rhodostoma* venom [71]. The study suggested that the discrepancy between the reported quantities is probably due to the lack of sensitivity in detecting other proteins in the whole venom. As for *O. hannah* venom, Tan et al. (2015) reported approximately 6% of LAAO from only two peptides [22], which is the same number of peptides detected in the current study. The difference in abundance is probably due to different quantitative analyses employed to determine the relative abundance. The sequence similarity of *O. hannah* LAAO peptides detected was observed in *C. rhodostoma* and *O. hannah* venoms, and the similarity with *C. rhodostoma* LAAO peptides was seen in *C. rhodostoma*, *B. schelegelii*, *T. stejnegeri*, *L. muta*, and *B. fasciatus* venoms. Several amine oxidase peptides were also observed in both venoms, with three peptides found in *C. rhodostoma* venom and one peptide in *O. hannah* venom.

##### NGF

This proteome analysis revealed 4% and 1% of nerve growth factors (NGFs) in *C. rhodostoma* and *O. hannah* venoms, respectively (Table 5, Figure 5 and Figure 6). In *C. rhodostoma* venom, a total of four peptides were discovered showing sequence similarity to venoms from *B. jararacussu*, *P. flavoviridis*, and *B. atrox*, whereas two peptides were found in *O. hannah* venom, with similarity to venoms from *W. aegyptia* and *O. hannah*. Snake venom nerve growth factors were first discovered serendipitously by Cohen and Levi-Montalcini [72]. In an effort to break down the nucleic acid portion of a purified component from a growth promoting secretion, *Agkistrodon piscivorus* venom was shown to be a significant inducer of nerve growth [72,73]. Since its discovery, numerous snake venom nerve growth factors have been purified and characterized from various snake venoms [74]. Reports have revealed that sv-NGFs are approximately 13 to 35 kDa, consisting of 10 to 20% of carbohydrates and are of great therapeutic interest for the treatment of neurodegenerative disease [75]. Apart from its growth promoting activity, sv-NGF has been proposed to possess vasculotoxin-like behavior, potentially to render the venom injection site more susceptible to facilitate infiltration of venom components into target tissues [76]. While sv-NGF has been established in Viperidae, Crotalinae, and Elapidae families, it is shown to occur in minute amounts (i.e., 0.1–0.5%) in several snake venoms [76,77]. Our findings here show a relatively higher amount than previously reported for both venoms [19,22].

##### PDE

Although PDEs are considered ubiquitously present in snake venoms, reports have described lower levels of PDE activity in elapids compared to crotalids and viperids [78]. In accordance, our analysis revealed 16% and 6% of phosphodiesterase (PDE) proteins in *C. rhodostoma* and *O. hannah* venoms, respectively (Table 5, Figure 5 and Figure 6). This class of toxins, also termed exonucleases, catalyzes the hydrolysis of phosphodiester bonds to release 5′-mononucleotides from the 3′ end of polynucleotides to provide nucleotide substrates to facilitate the activity of other toxins, such as 5′-nucleotidases [5,78]. In 1963, Russell et al. observed mean arterial pressure reduction and locomotor depression using PDE fractions from several snake venoms. Apart from this evidence of their potential function and as well as their hydrolytic properties, there is little information on the other pharmacological activities of these toxins [79]. The PDE peptides identified from *C. rhodostoma* venom showed similarity to peptides from *C. adamateus*, *C. horridus*, and *S. miliarius barbouri* venoms (Table 1 and Table 3). Likewise, *O. hannah* PDE peptides displayed similarity to *C. adamanteus* and *C. horridus*, as well as *O. okinavensis*, *M. fulvius*, and *M. tener* PDE peptides (Table 2 and Table 4). Interestingly, only minor amounts of PDEs were observed in the other proteomic analysis of King cobra and Malayan pit viper venoms [19,22,23,80].

##### Endonuclease

It is important to clarify that denominating endonucleases from nucleases has been a somewhat ambiguous endeavor. This is because endonucleolytic activity is an inherent property of venom PDEs (exonucleases). Endonucleases are made up of DNases that hydrolyze DNA, as well as RNases that hydrolyze RNA. Whereas, exonucleases are made up of PDEs that have the ability to hydrolyze both DNA and RNA [81]. To facilitate differentiation, parameters, such as the pH optima and metal ion requirement, were considered. To date, although this approach has provided some clarity, describing exclusive nuclease (endo and exo) activity is still considered a challenge. In our study, endonucleases were categorized from both venoms. Albeit in fairly low amounts, there was 3% in *C. rhodostoma* venom and 1% in *O. hannah* venom (Table 5, Figure 5 and Figure 6). Two peptides showing sequence similarity with *O. hannah* venom were discovered for both venoms, with an additional DNase-like protein showing sequence similarity with *C. adamanteus* found for *C. rhodostoma* venom (Table 2, Table 3 and Table 4). Thus far, no endonuclease proteins have been identified for these Malaysian specimens.

##### Nucleotidase

5′-Nucleotidase (5′-NTD) are high molecular weight glycoproteins and metalloenzymes ranging from 70 kDa to 100 kDa [77,79]. Primarily, 5′-NTDs endogenously liberates purines by selectively hydrolyzing 5′-nucleotidase to nucleosides [78,82]. Recent studies have indicated that 5′-NTDs play a crucial role in snake envenoming strategies of prey immobilization (via hypotension and paralysis) and prey digestion. This is because purine is the central component in all these strategies, whereby injection of exogenous nucleosides via venom and release of endogenous nucleosides via prey tissue, exert synchronous effects to bring about death. Thus, the ability of 5′-NTDs to provide a steady supply of purines has labelled them a multitoxin [79]. Though their biological functions have not been exhaustively studied, these enzymes are responsible for anticoagulant effects and inhibition of platelet aggregation in several snake venoms [83,84,85,86]. While ubiquitously distributed in snake venoms, 5′-NTD activity is more prominent in viperid venoms than elapid venoms. Our study reports 5% of 5′-NTDs in viperid *C. rhodostoma* venom and 2% in elapid *O. hannah* venom (Table 5, Figure 5 and Figure 6). Interestingly, the 5′-NTDs found in *O. hannah* venom were mainly ecto-nucleotidases showing sequence similarity to *M. tener* and *M. fulvius* (Table 2). Ecto-enzymes, such as ecto-5′-nucleotidases, are essentially membrane-embedded enzymes, with their active sites positioned on the exterior surface of the cell [78].

#### 2.5.2. Snake Venom Protein Classes in *C. rhodostoma* Venom

In our study, we have shown that *O. hannah* venom contains a relatively high abundance of disintegrins (SVMP) and minor amounts of CTLs, protein components that occur in almost all viper venoms. However, there were several protein families that were detected exclusively in the viper, *C. rhodostoma* venom, albeit in low abundance. These toxin families are described in the following section.

##### Low Abundance Proteins Only (<2%)

A total of three protein families with relative abundance of less than 2% each were discovered in *C. rhodostoma* venom. These proteins were from the aminopeptidase, glutaminyl-peptide cyclotransferase (QPCT), and Ankyrin (ANK; designated as ‘Others’) families (Table 5, Figure 5). The aminopeptidase protein family is considered one of several less well understood enzyme constituents of snake venom. Reports of aminopeptidase activity have been presented in elapidae and viperidae crude venoms as well as a crotalinae venom fraction [28]. However, although there has been evidence of aminopeptidase transcripts in several species of snake venom glands, aminopeptidase proteins have only been identified in three snake venom proteome studies [25,87,88]. Here, we report the proteomic identification of aminopeptidase peptides in the venom of crotalinae, *C. rhodostoma* for the first time (Table 3). The two identified peptides sequences showed similarity to the aminopeptidase sequence from *C. horridus* venom. Thus far, the role of aminopeptidases in snake venom has not been fully elucidated. However, analogous comparisons have been made with mammalian aminopeptidases, proposing their importance in regulating brain function and blood pressure [28,89]. Further suggestions include disrupting physiological processes though cleavage of oligopeptide N-terminals, degradation of host tissues, increasing permeability to venom components, or processing toxins components within the venom [28].

One glutaminyl-peptide cyclotransferase (QPCT) was detected in the *C. rhodostoma* proteome, constituting only 1% of the entire crude venom (Table 5, Figure 5). The peptide identified for this protein showed sequence similarity to QPCTs from the venom of another crotalinae, *A. contortrix contortrix* (Table 3). Remarkably, glutaminyl cyclases were found to be notably present in crotalines, but not viperine venoms. Studies that have identified this protein in snake venom proteomes include other crotalinaes, *C. durissus terrifcus*, *C. atrox*, and *C. purpureomaculatus* [32]. Interestingly, Zainal Abidin et al. (2016) reported the absence of this protein in another crotalinae, *T. wagleri*. However, the proteome of *T. wagleri* was reported to be somewhat atypical compared to other viperid species [32,62]. Additionally, transcripts have been reported in the venom glands of more crotalinaes, *S. catenus edwardsii* and *B. jararaca* [90,91,92,93]. QPCTs are considered to be another poorly understood protein component in snake venoms. Very few QPCTs have been purified or isolated and therefore not thoroughly evaluated [94]. Nevertheless, it has been suggested that QPCTs play a role in post-translational modification (PTM), whereby they catalyze the N-terminal pyroglutamate (pGlu) portion of toxins to release ammonia or water molecules. Atypical to toxins, QPCTs may be involved in toxin maturation, protection from exopeptidase degradation, and aiding accurate protein conformation [95].

One of the low abundance peptides identified in *C. rhodostoma* venom identified an unusual protein domain designated as Ankyrin repeat-containing protein showing sequence similarity to *A. contortrix contortrix* venom (Table 1, Figure 5). Interestingly, the Ankyrin protein was initially described as a 33 amino acid repeat found amongst a few proteins in *Drosophila melanogaster*, a common fruit fly. Later, it was termed a cytoskeletal protein that was made up almost entirely of these short repeats [96]. Since then, the Ankyrin (ANK) repeats have been found to occur in thousands of proteins in viruses, prokaryotes, and eukaryotes. No particular function has been attributed to the ANK repeats, however, there are suggestions of their involvement in mediating protein-protein interactions [97]. In venomous animals, the most prominent work on Ankyrin repeats has been reported in a black widow spider, *Latrodectus tredecimguttatus*, transcriptomics study. The Ankyrin repeats were grouped as a superfamily consisting of five subfamilies (mainly α-latrotoxins), which differed in the number and distribution of ANK domains present in individual toxin families. As the ANK repeats were predominantly abundant in the main neurotoxic proteins identified in the venom, ANKs were included as part of the neurotoxin family [98]. However, looking at the low protein score (Table S1) coupled with the identification of only a single ANK peptide, we believe that the protein does not play a significant role in the venom and may serve in other general non-toxic functions.

#### 2.5.3. Snake Venom Protein Classes in *O. hannah* Venom

One of the key differences between *C. rhodostoma* venom and *O. hannah* venom was the absence of 3FTx in the viper venom. This toxin family was identified as the most abundant protein family in the elapid venom. Other major protein families that were found exclusively in *O. hannah* venom were complement C3 homolog, Kunitz-type, and insulin, while several other low abundance families were also discovered in the venom, notably SVGF, hepatocyte growth factor (Table 5). The nine protein families only present in *O. hannah* venom are discussed below.

##### 3FTx

Three-finger toxins (3FTx) are a class of proteins that belong to the snake venom superfamily, with a conserved three finger like appearance in three dimensional structures. 3FTx are commonly found in venoms of elapid snakes and are known to be a major contributing lethal factor to Elapidae and also Hydrophidae venoms [99,100,101]. As expected, we found a total of 55 proteins (31%) belonging to this family from the Malaysian *O. hannah* venom, but not *C. rhodostoma* venom (Table 5, Figure 6). The majority of the 3FTxs detected belonged to the long-chain subfamily (65%), whereas the rest were appropriated to the short-chain and non-conventional subfamilies. All identified peptides showed similar sequences to Elapidae venoms; *N. kaouthia*, *N. oxiana*, *N. annulata annulata*, *N. haje haje*, *N. nivea*, *N. melanoleuca*, *N. naja*, *N. sputatrix*, *D. polylepis polylepis*, *D. viridis*, *B. multicinctus*, *B. flaviceps*, *A. superbus*, and *A. labialis* (Table 2 and Table 4). Interestingly, there was a sequence similarity detected with a Viperidae venom, *C. rhombeatus*, of a short chain 3FTx, neurotoxin-like protein 1. Evolution has allowed snakes to adapt to a variety of prey and although previously thought to be unique to elapidaes, studies have shown the presence of elapidae components across snake families, including a non-venomous *C. radiatus* gland secretion [102].

##### Complement C3 Homolog

The complement C3 homolog family consists of proteins interacting with components of the serum complement system that modulate the immune system. In venom, this group is a structural and functional analog of complement component C3b, the activated form of C3. The cobra venom factor (CVF) is one of the best studied proteins in this family [103]. It is a glycoprotein, ~150 kDa and is frequently composed of alpha, beta, and gamma chains. When introduced into the bloodstream, it stimulates complement activation and results in the consumption of the complement activity. The CVF protein is known to be specific to the cobra species of the Elapidae family. Although the function of the purified CVF has been demonstrated in vivo, the exact biological role of CVF in cobra venom is still not fully understood [103,104]. In our findings, we have categorized seven proteins (4%) in this family consisting of complement-depleting factor, Ophiophagus venom factor, and venom factors (Table 2 and Table 4, Figure 6). Despite being annotated as a venom factor of a cobra, the purified protein is known to be non-toxic [103].

##### Kunitz-Type

Kunitz-type is a family of toxins consisting of a Kunitz domain with a serine protease inhibiting function. The characteristic domains are made up of a conserved fold of about 60 amino acids and three disulfide bridges. The family is also known as the bovine pancreatic trypsin inhibitor (BPTI) and is naturally present across the animal taxa [105,106]. Similar to 3FTx, this toxin family has evolved due to gene duplication and positive selection. Originally, the role of Kunitz type toxins was to inhibit the function of serine protease, but, over the years, some have evolved to block ion channels, such as K^+^ and Ca^2+^. However, a few of these toxins have the novel ability of reserving their protease inhibitory function as well as ion channel modulatory function, and therefore acquiring a bifunctional status [107]. Here, we have identified six ‘original’ Kunitz-type proteins appearing to have retained their protease inhibitory function. The Kunitz-type protein made up 3% of the total venom (Table 2, Table 4 and Table 5, Figure 6). Although previous studies have isolated these serine protease inhibitors from Elapidae and Viperidae venoms [108], there were no peptides that matched the identity of a Kunitz-type protein for Malaysian *C. rhodostoma* venom.

##### Insulin

Over the years, the advancement of investigational techniques in protein chemistry have propelled the recognition of insulin as being a part of a large protein superfamily that is present in various types of organisms. This superfamily consists of insulin and related peptides, such as insulin-like peptides and insulin-like growth factors as well as relaxins [109]. One very exciting discovery is the first example of insulin in a venom and furthermore an invertebrate of a cone snail, *Conus geographus* [110]. In snake venom, particularly *O. hannah*, there has been evidence at the transcriptional level of venom glands [22,23] and proteomic level [80]. However, due to the lack of evidence in other snake venoms, the transcriptomic findings were classified as rare and thus, in need of more thorough or detailed investigation. Here, we would like to document the presence of five insulin-like growth factor 1 proteins identified for the first time at the proteomic level of Malaysian *O. hannah* venom, making up 3% of the total venom (Table 2 and Table 4, Figure 6). Previously, the lack of identification at the proteomic level was attributed to extremely minute amounts of proteins in the venom. Therefore, we believe that our discovery is noteworthy as almost all except one protein was identified through the concentrated molecular weight separation fractions compared to the crude shotgun method.

##### Low Abundant Proteins in *O. hannah* Venom (<2%)

Snake venom acetylcholinesterase (AChE) was first reported in 1938 when strong choline esterase activity was observed in three cobra species. Together with butyrylcholinesterase (BuChE), AChE makes up the snake venom cholinesterase family and functions to hydrolyze choline esters [111]. Studies have shown that *Bungarus fasciatus* venom contains high concentrations of AChE at approximately 8 mg/g of dry venom with strong activity reported at around 750,000 Ellman units/mg, designating this venom as the richest source of this enzyme. Interestingly, AChE activity has only been demonstrated in Elapidae venoms, but not in Viperidae or Crotalidae venoms [112]. Not surprisingly, we detected only two peptides belonging to the AChE family, one showing sequence similarity with *B. fasciatus* and the other a carboxylic ester hydrolase from *S. fasciata* (Table 2 and Table 4). No AChE peptides were recovered from *C. rhodostoma* venom. Neprilysin is a zinc-metalloendopeptidase with specificity to the bacterial zinc peptidase, thermolysin. Originally located in the rabbit kidney, the first observation of the protein in snake was in the form of a transcript in the saw scaled viper venom of *E. pyramidum leakeyi* [113,114]. Other neprilysin transcripts were reported in the Balinese and Malaysian king cobra. The latter study also reported the first proteomic evidence of neprilysin in Malaysian *O. hannah* venom [22]. In this study, we report the presence of one neprilysin (Table 2) compared to two identified by the other Malaysian sample. In mammals, neprilysin has been implicated in neuropeptide regulation, however, its role in venom has not been elucidated [115].

Another low abundant, but noteworthy, toxin family in *O. hannah* venom is the Ohanin/Vespryn family. This family is a relatively new family of snake venom proteins established in 2005. Ohanin, a novel protein with unusual molecular mass, was isolated from King cobra venom that displayed an uncharacteristically low content of cysteine residues (1%). Comparison of amino acid sequence revealed a 93% sequence similarity with Thai cobrin, an isoform from the monocled cobra, *Naja kaouthia*. However, to date, there is no literature available on Thai cobrin [116]. The proteins possess the PRY and SPRY domain (B30.2-like domain) sequences that are the trademark for vespryn proteins, but do not share sequence similarity to other proteins in the snake venom database. This feature, coupled with the low cysteine residue content, makes both proteins exclusive members of this toxin family. Ohanin has been synthesized as a precursor named prohanin, with a propeptide segment at the C-terminal segment that completes the SPRY domain [117]. Prohanin (THA 903) has shown strong pharmacological potential and is currently undergoing clinical trials for the treatment of chronic pain [118]. In this study, we identified ohanin from *O. hannah* venom and its isoform, Thaicobrin, from the *N. kaouthia* venom (Table 2 and Table 4), revealing a total of 1% of this protein family in the overall venom composition (Table 4, Figure 6).

The proteomic analysis of Malaysian king cobra in this study also discovered single peptides of snake venom growth factors (SVGFs): Hepatocyte-growth factor activator, macrophages colony-stimulating factor (MCSF), tumor necrosis factor receptor (grouped as SVGF), and one other peptide, PDGF/VEGF growth factor (Table 2 and Table 4, Figure 6). Historically, growth factors represent growth and cell proliferation. Since then, the attributes have evolved to include neutrophins, such as cell differentiation, transformation, synthesis secretions, motility, and death [119]. Over the years, hundreds of snake venom growth factors (SVGFs) have been isolated, studied, and deposited in the UniProt databank. This includes the abovementioned platelet-derived growth factor (PDGF), vascular endothelial growth factor (VEGF), tumor necrosis factor (TNF), hepatocyte growth factor-tyrosine kinase (HGF), insulin-like growth factor-binding protein (IGF), and nerve growth factor (NGF) that has been discussed throughout this proteomic analysis [77]. Remarkably, prior to this study, the hepatocyte growth factor activator protein was only identified at the proteomic level in Thai *O. hannah* [80]. The investigations of *O. hannah* species from Malaysia and Bali did not report the presence of this peptide in their proteomic analyses [21,22,23]. In this study, the hepatocyte growth factor protein was discovered using both proteomic approaches and constitutes approximately 1% of the entire venom (Table 2 and Table 4, Figure 6). The hepatocyte growth factor is known to stimulate development and migration of vascular endothelial cells. This particular growth factor has demonstrated accelerated glomerular restoration via capillary endothelial cell growth and capillary regeneration in experimental progressive glomerulonephritis [120]. The other two growth factors (TNF and MCSF) were also not identified in the Malaysian, Thai, or Balinese specimens [21,22,23,80]. However, the presence of VEGF proteins was reported in the Balinese and Thai proteomes [23,80].

The focus of this study was to provide comprehensive proteomic profiles of Malaysian CR and OH snakes by maximally identifying the proteins present within their venom composition. We utilized venom samples from three separate pools (species) from the northern region of Malaysia, which provides a robust statistical framework for general venom composition identification. Nonetheless, further work, including a broader area of sampling around Malaysia, is necessary for a more detailed appreciation of individual differences between species.

Finally, this venomic investigation has provided evidence of major venom proteins, including SVMP, PDE, SVSP, CTL/snaclec, PLA_2_, FMO, PLB, 5′-NTD, NGF, Endonuclease, and CRiSP, as well as minor venom proteins consisting of Aminopeptidase, QPCT, Trypsinogen, and Ankyrin repeats in *C. rhodostoma* venom. Likewise, major protein components from 3FTx, SVMP, CRiSP, PDE, PLA_2_, Complement C3, Kunitz-type, and Insulin, along with minor components from SVGF, FMO, 5′-NTD, CTL/snaclec, SVSP, AChE, Ohanin/Vespryn, NGF, Endonuclease, Neprilysin, PDGF/VEGF, and PLB were determined in *O. hannah* venom. At this point of the study, it is difficult to distinguish if the small numbers of low abundance proteins found in the venom are indeed novel species-specific proteins or rare proteins that are yet to be discovered in other snake species. Nevertheless, the data obtained from our global proteomic analysis has revealed the presence of inherent medically and pharmacologically important components within Malaysian *C. rhodostoma* and *O. hannah* crude venoms, possessing potential clinical utility as tools or as prototypes for drug design studies.

## 3. Conclusions

Using a combination of two proteomic approaches, shotgun and gel-filtration fractionation techniques, we have provided a holistic view of the complex Malaysian *C. rhodostoma* and *O. hannah* crude venom protein compositions. As a forethought, we would like to highlight that proteins from the insulin, CTL/snaclec, and SVGFs families were identified for the first time in *O. hannah* venom in a proteomics study. Proteins from the Aminopeptidase, QPCT, and Ankyrin families in *C. rhodostoma* venom were also reported for the first time. In addition, the total number of proteins identified in both venoms is higher than reported in previous proteomic studies. Looking at the discrepancies between previous studies and our study, this finding is perhaps not exhaustive, but definitely supplementary, to previous findings and data collection. Nonetheless, we reiterate that the discovery of these new proteins will significantly impact the knowledge of the venom components in both these species, which may be used as a reference for improved antivenom production, understanding pathological effects of envenoming, and the discovery of biologically active peptides with medical and biotechnological values.

## 4. Materials and Methods

### 4.1. Materials

#### 4.1.1. Snake Venom

*Calloselasma rhodostoma* and *Ophiophagus hannah* snake venoms were milked from adult snakes originating from the northern part of the west coast of Peninsular Malaysia (Perlis). Crude venoms were obtained from Mr. Zainuddin Ismail, a private snake enthusiast from Perlis. Manual milking was performed by placing the snake’s fangs on a parafilm-covered sterile container. Extracted venom samples were transported on ice to Monash University Malaysia (Sunway Campus). The venom samples were immediately frozen at −80 °C, lyophilized via a freeze-dryer, and stored at −20 °C until required. Quantification of the crude protein was carried out by performing a Bicinchoninic acid (BCA) assay as outlined by the manufacturer (Pierce Biotechnology, Rockford, IL, USA), using bovine serum albumin (BSA) as the standard.

#### 4.1.2. Columns, Drugs and Chemicals

The Superdex G-75 (10 mm × 300 mm) and Hi-trap G-25 (16 mm × 25 mm) were purchased from GE-Healthcare (Uppsala, Sweden), whereas the Zorbax 300SB-C18 (75 μm × 150 mm, 5 μm) was purchased from Agilent (Santa Clara, CA, USA). The chemicals and reagents used were of analytical grade, except LCMS grade materials from Fisher Scientific (Leicestershire, UK) for all LCMS work. Water used in this study was ultrapure.

### 4.2. Methods

#### 4.2.1. Sodium Dodedyl Sulfate-Polyacrylamide Gel Electrophoresis (SDS-PAGE)

Protein profiles of *C. rhodostoma* and *O. hannah* crude venom samples were assessed by SDS-PAGE according to Laemmli, 1970 [121]. Electrophoresis runs were carried out under reducing conditions in 12% resolving gels and 4% stacking gels in room temperature. Fractions were boiled at 95 °C for 5 min and allowed to cool to room temperature before being loaded onto the gel and run for approximately 60 min at 200 V in 1× SDS running buffer. The gels were fixed for 10 min in fixing solution and visualized through staining with Coomassie Blue R-350 (1 h or overnight) and de-staining with Coomassie de-stain (overnight). The molecular weights of protein bands were estimated using broad range SDS-PAGE molecular weight standards from Thermo Scientific (Rockford, AZ, USA).

#### 4.2.2. In-solution Protein Digestion

Crude venom solutions (Section 4.2.3) and gel-filtration protein fractions (Section 4.2.4) were prepared for identification using an in-solution digestion procedure according to the manufacturer’s instructions (Agilent Technologies, Santa Clara, CA, USA). Briefly, 25 μL of 100 mM ammonium bicarbonate (ABC), 25 μL of trifluoroethanol (TFE), and 1 μL of dithiothreitol (DTT) were added to concentrated protein crude venom samples and fractions. The mixture was briefly vortexed and incubated at 60 °C for 60 min, followed by incubation with 4 μL of iodoacetamide at 37 °C for another 60 min. 1 μL of DTT was added to the solution and incubated again for another 60 min. 100 mM ABC and trypsin [1:50 (*w*/*w*) enzyme-to-substrate ratio; approximately 500 ng] were then added to the solution and incubated at 37 °C for 18 h. At the end of the incubation, trypsin activity was terminated by adding formic acid and finally subjected to centrifugal vacuum evaporation to remove solvents and concentrate the sample simultaneously. Samples were monitored for 1 to 2 days until completely dry and subsequently stored at −20 °C until LC-MS/MS analysis.

#### 4.2.3. Identification of Crude Venom Proteins Using Shotgun LCMS/MS

Previously frozen lyophilized *C. rhodostoma* and *O. hannah* venoms were adjusted to 200 μg protein samples and subjected to in-solution tryptic digest protocol as described in Section 4.2.2. The crude venom samples from three separately pooled stocks were utilized for this study.

#### 4.2.4. Fractionation of *C. rhodostoma* and *O. hannah* Crude Venoms by Superdex G-75 Gel Filtration Chromatography

Freeze-dried crude venoms (10 mg) were dissolved in 0.01 M ammonium acetate (250 μL; pH 7.0) and centrifuged at 5000× *g* for 1 min to remove insoluble material. The clarified supernatant was subjected to gel filtration chromatography via Superdex G-75 pre-equilibrated and eluted with the same buffer at a flow rate of 0.5 mL/min. The fractionation was monitored using a fast protein liquid chromatography system (AKTA Prime Plus, GE Healthcare, Uppsala, Sweden) and carried out at room temperature. The chromatographic data were acquired and processed using Unicorn 5.20 Workstation software (GE Healthcare, Uppsala, Sweden). All fractions were pooled and subjected to buffer exchange using a Hi-Trap G-25 desalting column. Desalted fractions were concentrated and stored at −20 °C until required for mass spectrometry identification analysis. Samples from three separately pooled stocks were adjusted to 200 μg before being subjected to in-solution tryptic digest.

#### 4.2.5. LC-MS/MS Analysis of Crude Venom and Gel-Filtration Protein Fractions

Tryptic-digested peptides stored in −20 °C were reconstituted in 20 μL of 0.1% formic acid in water. 1 μL of peptide sample was injected on a nano C-18 column (see below) using the Agilent 1200 capillary nanoflow liquid chromatography system coupled to a 6550 iFunnel Q-TOF LC/MS mass spectrometer (Agilent) via a nano-electrospray (Nano-ESI) source. Digested peptide products were separated using the C18 300 Å Large Capacity Chip column (Zorbax 300SB-C18) that was pre-equilibrated with 0.1% formic acid in water (A) and eluted in a multi-gradient approach using 90% acetonitrile in 0.1% formic acid in water (B) at a flow rate of 4 μL/min. The method utilized a 47 min gradient starting with a 30 min ramping from 3% to 50% solution B, followed by a further increase of 50% to 95% solution B for 2 min, 95% solution B for 7 min, and finally a decrease from 95% to 3% solution B for another 8 min. BSA was used as a standard for reference. Mass spectrometry (MS/MS) was performed using these following settings: Gas temperature 300 °C; drying gas 5 L/min; capillary voltage of 2050 V; and fragmenter voltage 300 V. The auto MS/MS mode was used, with positive ion polarity setup. Acquisition ranges were 110–3000 *m*/*z* for MS scan and 50–3000 *m*/*z* for MS/MS scan.

#### 4.2.6. Data Analysis

Data analysis and protein identification by automated de novo sequencing was performed using PEAKS 8.0 software (Bioinformatics Solutions Inc., Waterloo, ON, Canada). MS/MS spectra were searched against the Swiss-Prot database (July 2017) by selecting all proteins from the Serpentes taxa subjected to the following parameters: Digestion Trypsin; maximum number of missed cleavages 3; fixed modification: carbamidomethylation; mass error tolerance (parent and fragment) 0.1 Da; false discovery rate (FDR) 1%; unique peptides 2; and peptide hit threshold (−10lgP) 20. Venom components were classified according to protein families and their relative abundances were calculated according to Zainal Abidin et al. (2016) [62] as follows:

$$\frac{\mathrm{number}\;\mathrm{of}\;\mathrm{proteins}\;\left(\mathrm{protein}\;\mathrm{family}\right)}{\mathrm{total}\;\mathrm{proteins}\;\mathrm{detected}\;\mathrm{using}\;\mathrm{LC}-\mathrm{MS}/\mathrm{MS}}\times 100\%$$

Relative abundance was expressed as percentages and represented in pie charts. A complete list of identified peptides and proteins is included as Supplementary Material (Tables S1–S4).

## Figures and Tables

**Table 1:** List of proteins identified from Malaysian *C. rhodostoma* venom using the shotgun-ESI-LC-MS/MS technique. Refer to Supplementary File (Table S1) for complete proteomic data and abbreviations lists.

Protein Family	Protein Description	No. of Proteins	Protein Accession No.	No. of Peptides	No. of Unique Peptides
5′-NTD	5′ nucleotidase (*P. flavoviridis*)	5	T2HRS9	4	2
	5-nucleotidase (*P. elegans*)		A0A077L6M5	4	2
	5′ nucleotidase (*P. flavoviridis*)		A0A077L7M9	4	2
	Snake venom 5′-nucleotidase (*A. piscivorus*)		A0A194APL9	3	2
	BATXNUC1 (*B. atrox*)		A0A1L8D667	3	2
CRiSP	Cysteine-rich secretory protein LCCL 2 (*C. adamanteus*)	1	A0A0F7ZEB6	2	2
CTL/snaclec	C-type lectin (*A. piscivorus leucostoma*)	12	G8FML6	4	4
	C-type lectin 2 (*A. piscivorus*)		A0A194APP2	6	6
	C-type lectin APL (*A. piscivorus piscivorus*)		P0DM36	4	4
	C-type lectin BjL (*B. jararaca*)		Q9PRY7	4	4
	C-type lectin PAL (*B. arietans*)		Q9PSN0	4	4
	Snaclec clone 2100755 (*D. acutus*)		Q8JIV8	3	3
	Snaclec rhodocetin subunit alpha (*C. rhodostoma*)		P81397	17	15
	Snaclec rhodocetin subunit beta (*C. rhodostoma*)		P81398	15	14
	Snaclec rhodocetin subunit gamma (*C. rhodostoma*)		D2YW39	6	4
	Snaclec rhodocetin subunit delta (*C. rhodostoma*)		D2YW40	6	6
	Snaclec rhodocytin subunit alpha (*C. rhodostoma*)		Q9I841	8	8
	Snaclec rhodocytin subunit beta (*C. rhodostoma*)		Q9I840	14	13
FMO	Amine oxidase (*O. okinavensis*)	2	T2HQ57	18	2
	L-amino-acid oxidase (*C. rhodostoma*)		P81382	53	34
PDE	Cadam10_PDE-1 (*C. adamanteus*)	7	A0A1W7RBB0	6	2
	Ectonucleotide pyrophosphatase/phosphodiesterase 3 (*C. horridus*)		T1DJT5	6	2
	Phosphodiesterase (*C. horridus*)		T1D6P7	6	3
	Phosphodiesterase (*C. adamanteus*)		A0A0F7Z2Q3	6	3
	Phosphodiesterase (*S. miliarius barbouri*)		A0A194AS02	6	2
	Venom phosphodiesterase 1 (*C. adamanteus*)		J3SEZ3	6	3
	Venom phosphodiesterase 2 (*C. adamanteus*)		J3SBP3	6	3
PLA_2_	Acidic phospholipase A_2_ H1E6 (*C. rhodostoma*)	6	Q9PVF2	11	10
	Acidic phospholipase A_2_ S1E6-b (*C. rhodostoma*)		Q9PVF0	6	5
	Acidic phospholipase A_2_ Ts-A4 (*T. stejnegeri*)		Q6H3C7	11	10
	Basic phospholipase A_2_ W6D49 (*C. rhodostoma*)		Q9PVF4	21	6
	Phospholipase A_2_ (*C. rhodostoma*)		A0A0H3U266	23	6
	Phospholipase A_2_ (*T. sabahi*)		A0A0H3U232	3	2
PLB	Phospholipase B (*O. okinavensis*)	2	T2HQ75	3	2
	Phospholipase B (*P. regius*)		A0A098LY74	5	3
QPCT	Glutaminyl-peptide cyclotransferase (*A. contortrix contortrix*)	1	A0A1W7RH88	2	2
SVMP	Metalloprotease, mRNA (*G. intermedius*)	5	A0A0C4ZNF1	5	2
	Snake venom metalloproteinase kistomin (*C. rhodostoma*)		P0CB14	86	86
	Zinc metalloproteinase/disintegrin (*C. rhodostoma*)		P30403	38	36
	Zinc metalloproteinase-disintegrin-like halysase (*G. halys*)		Q8AWI5	4	2
	Zinc metalloproteinase-disintegrin-like HV1 (*P. flavoviridis*)		Q90ZI3	4	2
SVSP	Serine protease 1 (*P. elegans*)	4	A0A077LA46	3	2
	Thrombin-like enzyme ancrod (*C. rhodostoma*)		P26324	21	20
	Thrombin-like enzyme ancrod-2 (*C. rhodostoma*)		P47797	17	13
	Thrombin-like enzyme elegaxobin-2 (*P. elegans*)		P84787	3	2
Trypsinogen	Trypsinogen homolog (*B. jararaca*)	1	Q9PUF3	3	3
Others	Ankyrin repeat-containing protein (*A. contortrix contortrix*)	1	A0A1W7RJF1	2	2
	**TOTAL PROTEINS**	47			

Abbreviations: 5′-NTD: 5′-nucleotidase; CRiSP: Cysteine-rich secretory protein; CTL: C-type lectin; FMO: Flavin monoamine oxidase; PDE: Phosphodiesterase; PLA_2_: Phospholipase A_2_; PLB: Phospholipase B; QPCT: Glutaminyl-peptide cyclotransferase; SVMP: Snake venom metalloproteinase; SVSP: Snake venom serine protease.

**Table 2:** List of proteins identified in Malaysian *O. hannah* venom via the shotgun-ESI-LC-MS/MS technique. Refer to Supplementary File (Table S2) for complete proteomic data and abbreviations lists.

Protein Family	Protein Description	No. of Proteins	Protein Accession No.	No. of Peptides	No. of Unique Peptides
3FTx	Alpha-elapitoxin-Dpp2d (*D. polylepis polylepis*)	23	C0HJD7	2	2
	Beta-cardiotoxin CTX27 (*O. hannah*)		Q69CK0	11	3
	Haditoxin (*O. hannah*)		A8N286	3	3
	Long neurotoxin 1 (*O. hannah*)		P01387	8	2
	Long neurotoxin 1 (*N. nivea*)		P01390	2	2
	Long neurotoxin 2 (*O. hannah*)		P01386	7	2
	Long neurotoxin 4 (*O. hannah*)		P80156	2	2
	Long neurotoxin LNTX1 (*O. hannah*)		Q2VBP8	4	2
	Long neurotoxin LNTX-2 homolog (*O. hannah*)		A8N285	8	2
	Long neurotoxin LNTX37 (*O. hannah*)		Q2VBP3	3	2
	Long neurotoxin OH-5 (*O. hannah*)		P80965	2	2
	Long neurotoxin OH-37 (*O. hannah*)		Q53B59	7	7
	Long neurotoxin OH-55 (*O. hannah*)		Q53B58	13	6
	Long neurotoxin OH-56 (*O. hannah*)		Q53B57	4	4
	Long neurotoxin OH-57 (*O. hannah*)		Q53B56	3	3
	Neurotoxin-like protein 1 (*C. rhombeatus*)		P84716	2	2
	Short neurotoxin OH-35 (*O. hannah*)		Q53B49	5	5
	Weak toxin DE-1 (*O. hannah*)		P01412	36	29
	Weak toxin DE-1 homolog 1 (*O. hannah*)		Q69CJ8	2	2
	Weak neurotoxin OH-72 (*O. hannah*)		Q53B61	4	3
	Weak neurotoxin WNTX33 (*O. hannah*)		Q2VBN3	23	16
	Weak neurotoxin WNTX34 (*O. hannah*)		Q2VBN2	5	4
	Uncharacterized protein (*O. hannah*)—Toxin activity		V8N3G9	5	5
5′-NTD	Ecto-5′-nucleotidase (*M. tener*)	3	A0A194AS98	3	2
	Ecto-5′-nucleotidase 1 (*M. fulvius*)		U3FYP9	3	2
	Ecto-5′-nucleotidase 1a (*M. fulvius*)		A0A0F7YZM6	3	2
AChE	Acetylcholinesterase (*B. fasciatus*)	1	Q92035	5	5
CC3H	*O.* venom factor (*O. hannah*)	1	I2C090	8	6
CRiSP	Cysteine-rich venom protein (*T. stejnegeri*)	4	P60623	2	2
	Cysteine-rich venom protein ablomin (*G. blomhoffii*)		Q8JI40	2	2
	Cysteine-rich venom protein mossambin (*N. mossambica*)		P0DL16	2	2
	Cysteine-rich venom protein ophanin (*O. hannah*)		Q7ZT98	13	12
CTL/snaclec	Snaclec rhodocetin subunit beta (*C. rhodostoma*)	1	P81398	2	2
Endonuclease	Endonuclease domain-containing 1 protein (*O. hannah*)	1	V8NCE3	2	2
FMO	Amine oxidase (*O. hannah*)	3	V8N3Q9	29	2
	L-amino-acid oxidase (*O. hannah*)		P81383	16	16
	L-amino-acid oxidase (*C. rhodostoma*)		P81382	8	7
Insulin	Insulin-like growth factor 1 (*P. guttatus*)	1	I6TIH6	7	6
Kunitz-type	Kunitz-type serine protease inhibitor TCI (*O. hannah*)	5	B6RLX2	13	11
	Kunitz-type serine protease inhibitor Vur-Kin (*V. renardi*)		P0DKL8	2	2
	Kunitz trypsin inhibitor protein 2 (*V. renardi*)		S4S375	2	2
	Venom Kunitz trypsin inhibitor protein 2 (*V. renardi*)		S4S374	2	2
	Uncharacterized protein (*O. hannah*)—Peptidase inhibitor activity		V8NAQ0	2	2
Neprilysin	Neprilysin (*B. irregularis*)	1	A0A0B8RU83	5	2
NGF	Venom nerve growth factor (*W. aegyptia*)	2	V9I1F9	2	2
	Uncharacterized protein (*O. hannah*)		V8N4D8	2	2
Ohanin/Vespryn	Ohanin (*O. hannah*)	1	P83234	5	5
PDE	Ectonucleotide pyrophosphatase/phosphodiesterase 3 (*M. fulvius*)	3	U3FAB3	9	7
	Phosphodiesterase (*M. fulvius*)		A0A0F7YYZ8	9	7
	Phosphodiesterase (*M. tener*)		A0A194ARD7	9	7
PDGF/VEGF	Uncharacterized protein (*O. hannah*)	1	V8P6V3	5	5
PLA_2_	Acidic phospholipase A_2_ 2 (*O. hannah*)	2	Q9DF33	4	4
	Basic phospholipase A_2_ (*C. rhodostoma*)		Q9PVF4	2	2
SVGF	Hepatocyte growth factor activator (*O. hannah*)	1	V8NIY2	2	2
SVMP	Metalloproteinase (type III) 2 (*Hypsiglena* sp.)	21	A0A098M156	3	2
	Metalloproteinase type III 14a (*Hypsiglena* sp.)		A0A098M221	3	2
	Metalloproteinase type III 14b (*Hypsiglena* sp.)		A0A098M219	3	2
	Metalloproteinase (type III) 14c (*Hypsiglena* sp.)		A0A098M223	3	2
	Metalloproteinase type III 2 (*M. tener*)		A0A194AS47	8	2
	Scutellatease-1 (*O. scutellatus*)		B5KFV5	4	3
	Snake venom metalloproteinase BaP1 (*B. asper*)		P83512	3	2
	Snake venom metalloproteinase BjussuMP-2 (*B. jararacussu*)		Q7T1T4	3	2
	Snake venom metalloproteinase BmooMPalpha-I (*B. moojeni*)		P85314	3	2
	Snake venom metalloproteinase bothrojaractivase (*B. jararaca*)		P0C7A9	3	2
	Snake venom metalloproteinase kistomin (*C. rhodostoma*)		P0CB14	14	14
	Snake venom metalloproteinase leucurolysin-A (*B. leucurus*)		P84907	3	2
	Zinc metalloproteinase/disintegrin (*B. asper*)		Q072L5	3	2
	Zinc metalloproteinase/disintegrin (*B. insularis*)		Q5XUW8	3	2
	Zinc metalloproteinase/disintegrin (*C. rhodostoma*)		P30403	5	5
	Zinc metalloproteinase-disintegrin-like atrase-A (*N. atra*)		D5LMJ3	4	2
	Zinc metalloproteinase-disintegrin-like atrase-B (*N. atra*)		D6PXE8	5	2
	Zinc metalloproteinase-disintegrin-like BjussuMP-1 (*B. jararacussu*)		Q1PHZ4	3	2
	Zinc metalloproteinase-disintegrin-like kaouthiagin (*N. atra*)		D3TTC1	8	4
	Zinc metalloproteinase-disintegrin-like MTP9 (*D. coronoides*)		F8RKV9	6	2
	Zinc metalloproteinase-disintegrin-like ohanin (*O. hannah*)		A3R0T9	5	5
SVSP	Alpha and beta fibrinogenase OhS1 (*O. hannah*)	1	A8QL56	2	2
	**TOTAL PROTEINS**	76			

Abbreviations: 3FTx: Three-finger toxin; 5′-NTD: 5′-nucleotidase; AChE: Acetylcholinesterase; CC3H: Complement C3 homolog; CRiSP: Cysteine-rich secretory protein; CTL: C-type lectin; FMO: Flavin monoamine oxidase; NGF: Nerve growth factor; PDE: Phosphodiesterase; PDGF/VEGF: Platelet-derived growth factor/vascular endothelial growth factor; PLA_2_: Phospholipase A_2_; SVGF: Snake venom growth factor; SVMP: Snake venom metalloproteinase; SVSP: Snake venom serine protease.

**Table 3:** List of proteins identified from Malaysian *C. rhodostoma* venom using the GF-LC-MS/MS technique. Refer to Supplementary File (Table S3) for complete proteomic data and abbreviations lists.

Protein Family	Protein Description	No. of Proteins	Protein Accession No.	No. of Peptides	No. of Unique Peptides
5′-NTD	Snake venom 5′-nucleotidase (*B. irregularis*)	4	A0A0B8RXZ9	12	3
	5′-nucleotidase (*P. flavoviridis*)		T2HRS9	4	4
	5′-nucleotidase (*P. elegans*)		A0A077L6M5	5	4
	5′-nucleotidase (*P. flavoviridis*)		A0A077L7M9	4	4
Aminopeptidase	Aminopeptidase (*C. horridus*)	2	T1DNX8	4	4
	Aminopeptidase (*C. horridus*)		A0A0K8S2L4	4	4
CRiSP	Cysteine-rich secretory protein LCCL … 2 (*C. adamanteus*)	1	A0A0F7ZEB6	9	9
CTL/snaclec	Snaclec alboaggregin-D subunit alpha (*T. albolabris*)	8	P0DM38	4	3
	Snaclec rhodocetin subunit alpha (*C. rhodostoma*)		P81397	58	57
	Snaclec rhodocetin subunit beta (*C. rhodostoma*)		P81398	54	53
	Snaclec rhodocetin subunit gamma (*C. rhodostoma*)		D2YW39	25	23
	Snaclec rhodocetin subunit delta (*C. rhodostoma*)		D2YW40	22	22
	Snaclec rhodocytin subunit alpha (*C. rhodostoma*)		Q9I841	12	11
	Snaclec rhodocytin subunit beta (*C. rhodostoma*)		Q9I840	10	8
	C-type lectin 12a (*A. contortrix contortrix*)		A0A1W7RJZ7	7	7
Endonuclease	Endonuclease domain-containing protein (*O. hannah*)	3	U3FCT9	2	2
	Endonuclease domain-containing protein (*O. hannah*)		V8NCE3	2	2
	Deoxyribonuclease-2-alpha-like (*C. adamanteus*)—DNase		J3RZ14	4	4
FMO	Amine oxidase (*O. okinavensis*)	8	T2HQ57	40	4
	Amine oxidase (*P. regius*)		A0A098LWS4	12	3
	Amine oxidase (*P. guttatus*)		A0A098LX00	11	2
	L-amino-acid oxidase (*C. rhodostoma*)		P81382	147	99
	L-amino acid oxidase (*B. schlegelii*)		A0A024BTN9	46	10
	L-amino-acid oxidase (*T. stejnegeri*)		Q6WP39	19	2
	L-amino-acid oxidase (*L. muta*)		J7H670	15	2
	L-amino-acid oxidase (*B. fasciatus*)		A8QL52	10	2
NGF	Venom nerve growth factor (*B. jararacussu*)	4	Q90W38	3	2
	Nerve growth factor (*P. flavoviridis*)		B1Q3K2	5	5
	Nerve growth factor (*P. flavoviridis*)		A0A077L854	5	5
	BATXNGF1 (*B. atrox*)		A0A1L8D608	3	2
PDE	Cadam 10_PDE-1 (*C. adamanteus*)	18	A0A1W7RBB0	12	2
	Ectonucleotide pyrophosphatase/phosphodiesterase 3 (*M. fulvius*)		U3FAB3	5	2
	Ectonucleotide pyrophosphatase/phosphodiesterase 3 (*C. horridus*)		T1DJT5	12	3
	Phosphodiesterase (*P. flavoviridis*)		T2HQA0	17	2
	Phosphodiesterase (*P. flavoviridis*)		T2HRT4	17	2
	Phosphodiesterase (*P. flavoviridis*)		T2HPD6	17	2
	Phosphodiesterase (*P. flavoviridis*)		T2HP62	17	2
	Phosphodiesterase (*M. fulvius*)		A0A0F7YYZ8	5	2
	Phosphodiesterase (*M. tener*)		A0A194ARD7	5	2
	Phosphodiesterase (*M. fulvius*)		A0A0F7YYZ8	3	2
	Phosphodiesterase (*C. adamanteus*)		A0A0F7Z2Q3	12	3
	Phosphodiesterase (*C. horridus*)		T1D6P7	12	3
	Phosphodiesterase (*S. miliarius barbouri*)		A0A194AS02	12	3
	Phosphodiesterase (*M. tener*)		A0A194ARD7	3	2
	Phosphodiesterase (*M. rudis*)		A0A141DWM1	4	3
	Phosphodiesterase (*M. lebetina*)		W8E7D1	4	2
	Venom phosphodiesterase 1 (*C. adamanteus*)		J3SEZ3	12	2
	Venom phosphodiesterase 2 (*C. adamanteus*)		J3SBP3	12	2
PLA_2_	Acidic phospholipase A_2_ (*C. rhodostoma*)	10	Q9PVF0	4	3
	Acidic phospholipase A_2_ (*C. rhodostoma*)		Q9PVF1	16	4
	Acidic phospholipase A_2_ (*C. rhodostoma*)		Q9PVF2	57	54
	Acidic phospholipase A_2_ (*C. rhodostoma*)		Q9PVE9	6	2
	Acidic phospholipase A_2_ (*O. hannah*)		Q9DF33	5	5
	Acidic phospholipase A_2_ (*T. stejnegeri*)		Q6H3C7	34	32
	Basic phospholipase A_2_ W6D49 (*C. rhodostoma*)		Q9PVF4	25	11
	Phospholipase A_2_ (*C. rhodostoma*)		A0A0H3U266	41	19
	Phospholipase A_2_ (*C. atrox*)		A0A1J0R081	5	2
	Phospholipase A_2_ 1c (*C. horridus*)		A0A0K8RZ17	5	2
PLB	Phospholipase B (*C. adamanteus*)	6	F8S101	16	5
	Phospholipase B (*C. adamanteus*)		A0A0F7Z632	16	5
	Phospholipase B (*P. regius*)		A0A098LY74	8	2
	Phospholipase B (*P. guttatus*)		A0A098LWY9	4	2
	Phospholipase B (*P. flavoviridis*)		T2HP68	7	5
	Phospholipase B (*P. elegans*)		A0A077L7E7	7	5
SVMP	AAV1 protein (*D. acutus*)	23	A0A0M4MEY5	4	2
	BATXSVMPIII (*B. atrox*)		A0A1L8D5X9	3	2
	Disintegrin triflavin (*P. flavoviridis*)		P21859	8	6
	Metalloprotease, mRNA (*G. intermedius*)		A0A0C4ZNF1	8	2
	Metalloproteinase type III 2 (*M. tener*)		A0A194AS47	3	2
	Metalloproteinase type III 2 (*M. tener*)		A0A194AR91	7	3
	Metalloproteinase type III 2 (*Hypsiglena* sp.)		A0A098M156	2	2
	Metalloproteinase type III 14c (*Hypsiglena* sp.)		A0A098M223	2	2
	Metalloproteinase type III 5 (*Hypsiglena* sp.)		A0A098M215	2	2
	p-III snake venom metalloprotease (*M. ikaheca*)		A0A024AXX7	5	2
	Snake venom metalloproteinase kistomin (*C. rhodostoma*)		P0CB14	198	197
	SVMP-disintegrin-like mocarhagin (*N. mossambica*)		Q10749	8	4
	SVMP-Hop-15 (*H. bungaroides*)		R4FJZ4	5	2
	Zinc metalloproteinase/disintegrin (*C. rhodostoma*)		P30403	58	58
	Zinc metalloproteinase-disintegrin-like agkihagin (*D. acutus*)		Q1PS45	8	2
	Zinc metalloproteinase-disintegrin-like atragin (*N. atra*)		D3TTC2	4	2
	Zinc metalloproteinase-disintegrin-like atrase-A (*N. atra*)		D5LMJ3	4	2
	Zinc metalloproteinase-disintegrin-like BfMP (*B. fasciatus*)		A8QL48	7	7
	Zinc metalloproteinase-disintegrin-like BmMP (*B. multicinctus*)		A8QL49	7	7
	Zinc metalloproteinase-disintegrin-like cobrin (*N. kaouthia*)		Q9PVK7	5	2
	Zinc metalloproteinase-disintegrin-like halysase (*G. halys*)		Q8AWI5	8	2
	Zinc metalloproteinase-disintegrin-like HV1 (*P. flavoviridis*)		Q90ZI3	9	2
	Zinc metalloproteinase-disintegrin-like ohanin (*O. hannah*)		A3R0T9	12	12
SVSP	Snake venom serine protease catroxase (*C. atrox*)	12	Q8QHK2	3	2
	Snake venom serine proteinase (*C. adamanteus*)		F8S116	3	2
	Serine protease (*P. flavoviridis*)		A0A077L6P3	12	2
	Serine proteinase 3 (*A. piscivorus*)		A0A194APA8	3	2
	Serine proteinase 3a (*A. contortrix contortrix*)		A0A1W7RJX1	3	2
	Serine proteinase 4 (*A. contortrix contortrix*)		A0A1W7RJU2	3	2
	Serine proteinase 9 (*A. contortrix contortrix*)		A0A1W7RJU0	3	2
	Serine proteinase 10 (*C. horridus*)		T1DH10	5	2
	Serine proteinase 12a (*A. piscivorus*)		A0A194APD3	3	2
	Thrombin-like enzyme ancrod (*C. rhodostoma*)		P26324	64	59
	Thrombin-like enzyme ancrod-2 (*C. rhodostoma*)		P47797	39	37
	Thrombin-like protein (*A. piscivorus leucostoma*)		E9NX14	3	2
	**TOTAL PROTEINS**	99			

Abbreviations: 5′-NTD: 5′-nucleotidase; CRiSP: Cysteine-rich secretory protein; CTL: C-type lectin; FMO: Flavin monoamine oxidase; NGF: Nerve growth factor; PDE: Phosphodiesterase; PLA_2_: Phospholipase A_2_; PLB: Phospholipase B; SVMP: Snake venom metalloproteinase; SVSP: Snake venom serine protease.

**Table 4:** List of proteins identified from Malaysian *O. hannah* venom using the GF-LC-MS/MS technique. Refer to Supplementary File (Table S4) for complete proteomic data and abbreviations lists.

Protein Family	Protein Description	No. of Proteins	Protein Accession No.	No. of Peptides	No. of Unique Peptides
3FTx	Alpha-cobratoxin (*N. kaouthia*)	51	P01391	4	2
	Alpha-elapitoxin-Dpp2d (*D. polylepis polylepis*)		C0HJD7	3	2
	Alpha-elapitoxin-Dv2a (*D. viridis*)		P01395	3	2
	Alpha-elapitoxin-Nno2a (*N. oxiana*)		P01382	5	4
	Alpha-elapitoxin-Oh2b (*O. hannah*)		P82662	15	4
	Haditoxin (*O. hannah*)		A8N286	16	16
	Kappa-6-bungarotoxin (*B. multicinctus*)		Q9W729	3	2
	Long neurotoxin 1 (*A. superbus*)		A8S6A8	3	2
	Long neurotoxin 1 (*N. annulata annulata*)		P34074	9	2
	Long neurotoxin 1 (*N. haje haje*)		P25674	9	4
	Long neurotoxin 1 (*N. nivea*)		P01390	7	4
	Long neurotoxin 1 (*O. hannah*)		P01387	26	6
	Long neurotoxin 2 (*O. hannah*)		P01386	5	5
	Long neurotoxin 2 (*N. melanoleuca*)		P01388	8	3
	Long neurotoxin 3 (*O. hannah*)		P07526	5	2
	Long neurotoxin 4 (*O. hannah*)		P80156	14	3
	Long neurotoxin LlLong (*L. laticaudata*)		Q7T2I3	3	2
	Long neurotoxin OH-17 (*O. hannah*)		Q53B54	8	2
	Long neurotoxin OH-37 (*O. hannah*)		Q53B59	20	9
	Long neurotoxin OH-55 (*O. hannah*)		Q53B58	44	21
	Long neurotoxin OH-56 (*O. hannah*)		Q53B57	18	15
	Long neurotoxin OH-57 (*O. hannah*)		Q53B56	7	5
	Long neurotoxin LNTX1 (*O. hannah*)		Q2VBP8	13	5
	Long neurotoxin LNTX-2 (*O. hannah*)		A8N285	14	3
	Long neurotoxin LNTX8 (*O. hannah*)		Q2VBP6	21	3
	Long neurotoxin LNTX22 (*O. hannah*)		Q2VBP5	5	3
	Non-conventional three finger toxin isoform (*B. flaviceps*)		D5J9P9	4	2
	Oxiana weak toxin (*N. oxiana*)		P85520	3	3
	Short neurotoxin OH-35 (*O. hannah*)		Q53B49	5	5
	Three-finger toxin (*B. multicinctus*)		E2IU12	2	2
	Three-finger toxin (*B. multicinctus*)		E2IU14	2	2
	Weak neurotoxin (*N. N.*)		P29181	2	2
	Weak neurotoxin OH-72 (*O. hannah*)		Q53B61	13	10
	Weak neurotoxin WNTX33 (*O. hannah*)		Q2VBN3	65	55
	Weak neurotoxin WNTX34 (*O. hannah*)		Q2VBN2	13	10
	Weak neurotoxin 7 (*N. N.*)		P29181	4	3
	Weak neurotoxin 10 (*N. sputatrix*)		Q802B2	2	2
	Weak toxin CM-11 (*N. haje haje*)		P01401	3	3
	Weak toxin DE-1 (*O. hannah*)		P01412	117	110
	Weak toxin DE-1 homolog 1 (*O. hannah*)		Q69CJ8	12	12
	Weak toxin S4C11 (*N. melanoleuca*)		P01400	3	3
	Putative long neurotoxin (*A. labialis*)		B2BRQ8	3	2
	Putative long neurotoxin (*A. labialis*)		B2BRQ9	3	2
	Putative long neurotoxin (*A. labialis*)		B2BRR0	3	2
	Putative long neurotoxin (*A. labialis*)		B2BRR1	3	2
	Putative long neurotoxin (*A. labialis*)		B2BRR2	3	2
	Putative long neurotoxin (*A. labialis*)		B2BRR3	3	2
	Putative long neurotoxin (*A. labialis*)		B2BRR6	3	2
	Putative long neurotoxin (*A. labialis*)		B2BRR8	3	2
	Uncharacterized protein (*O. hannah*)—Toxin activity		V8N389	7	5
	Uncharacterized protein (*O. hannah*)—Toxin activity		V8N3G9	5	5
AChE	Carboxylic ester hydrolase (*S. fasciata*)	1	R4FKE6	2	2
CC3H	Complement-depleting factor (*O. hannah*)	7	A8QL55	8	8
	*O.* venom factor (*O. hannah*)		I2C090	34	23
	Venom factor (*C. horridus*)		T1E3W8	2	2
	Venom factor (*Sistrurus catenatus tergeminus*)		A0A194APJ4	2	2
	Venom factor (*Agkistrodon piscivorus*)		A0A194ARG1	2	2
	Venom factor (*C. adamanteus*)		A0A0F7Z1I7	2	2
	Venom factor (*C. adamanteus*)		J3S836	2	2
CRiSP	Cysteine-rich venom protein (*P. olfersii*)	13	Q09GJ9	3	2
	Cysteine-rich venom protein mossambin (*N. mossambica*)		P0DL16	9	2
	Cysteine-rich venom protein ophanin (*O. hannah*)		Q7ZT98	44	14
	Cysteine-rich venom protein TRI1 (*T. biscutatus*)		Q2XXP4	4	2
	Cysteine-rich secretory protein (*O. okinavensis*)		T2HPR8	7	2
	Cysteine-rich secretory protein (*P. chamissonis*)		A0A0B4SXI8	2	2
	Cysteine-rich secretory protein Az-CRP (*A. feae*)		F2Q6E3	6	2
	Cysteine-rich secretory protein Bc-CRP (*B. schlegelii*)		F2Q6E4	7	2
	Cysteine-rich secretory protein Bc-CRPa (*B. candidus*)		F2Q6G3	3	3
	Cysteine-rich secretory protein Bc-CRPb (*B. candidus*)		F2Q6G2	2	2
	Cysteine-rich secretory protein 1b (*B. irregularis*)		A0A0B8RZW7	3	2
	Cysteine-rich secretory protein 1c (*B. irregularis*)		A0A0B8RYV8	3	2
	Uncharacterized protein (*O. hannah*)		V8N8B4	35	5
CTL/snaclec	Snaclec rhodocetin subunit alpha (*C. rhodostoma*)	1	P81397	2	2
Endonuclease	Endonuclease domain-containing protein (*O. hannah*)	2	V8NCE3	3	2
	Endonuclease domain-containing protein (*O. hannah*)		V8N4Y2	3	2
FMO	Amine oxidase (*O. hannah*)	2	V8N3Q9	52	8
	L-amino-acid oxidase (*O. hannah*)		P81383	59	15
Insulin	Insulin-like growth factor 1 (*P. guttatus*)	5	I6TIH6	19	15
	Insulin-like growth factor I (*O. hannah*)		V8NHJ8	7	5
	Insulin-like growth factor 1 (*C. helena*)		I6S9K4	7	2
	Insulin-like growth factor 1 (*T. elegans*)		I6T666	9	9
	Uncharacterized protein (*O. hannah*)—Hormone activity		V8N2J2	4	4
Kunitz-type	Kunitz-type serine protease inhibitor (*O. hannah*)	6	P82966	7	3
	Kunitz-type serine protease inhibitor TCI (*O. hannah*)		B6RLX2	31	27
	Kunitz-type serine protease inhibitor Vur-KIn (*V. renardi*)		P0DKL8	4	3
	Kunitz trypsin inhibitor protein 2 (*V. renardi*)		S4S375	4	3
	Venom Kunitz trypsin inhibitor protein 2 (*V. renardi*)		S4S374	4	3
	Uncharacterized protein (*O. hannah*)—Peptidase inhibitor activity		V8NAQ0	4	4
NGF	Uncharacterized protein (*O. hannah*)	1	V8N4D8	2	2
Ohanin/Vespryn	Ohanin (*O. hannah*)	2	P83234	22	17
	Thaicobrin (*N. kaouthia*)		P82885	2	2
PDE	Cadam 10_PDE-1 (*C. adamanteus*)	11	A0A1W7RBB0	3	2
	Ectonucleotide pyrophosphatase/phosphodiesterase 3 (*M. fulvius*)		U3FAB3	11	11
	Ectonucleotide pyrophosphatase/phosphodiesterase 3 (*C. horridus*)		T1DJT5	3	2
	Phosphodiesterase (*M. tener*)		A0A194ARD7	11	11
	Phosphodiesterase (*M. fulvius*)		A0A0F7YYZ8	11	11
	Phosphodiesterase (*C. horridus*)		T1D6P7	3	2
	Phosphodiesterase (*C. adamanteus*)		A0A0F7Z2Q3	3	2
	Phosphodiesterase (*O. okinavensis*)		U3TBJ5	3	2
	Phosphodiesterase (*O. okinavensis*)		U3TAI7	3	2
	Venom phosphodiesterase 1 (*C. adamanteus*)		J3SEZ3	3	2
	Venom phosphodiesterase 2 (*C. adamanteus*)		J3SBP3	3	2
PDGF/VEGF	Uncharacterized protein (*O. hannah*)	1	V8P6V3	7	7
PLA_2_	Acidic phospholipase A_2_ 2 (*O. hannah*)	8	Q9DF33	54	49
	Acidic phospholipase A_2_ (*O. hannah*)		Q9DF56	20	17
	Acidic phospholipase A_2_ KBf-grIB (*B. fasciatus*)		P0C551	2	2
	Phospholipase A_2_ 12 (*M. fulvius*)		U3FVG1	5	2
	Phospholipase A_2_ 23 (*M. fulvius*)		U3FVF5	5	2
	PLA_2_ (IB)-Tri1 (*T. biscutatus*)		A7X418	5	3
	Secretory phospholipase A_2_ (Group 10) (*O. hannah*)		V8PDP0	2	2
	Uncharacterized protein (*O. hannah*)		V8N1D8	2	2
PLB	Phospholipase B-like 1 (*O. hannah*)	1	V8ND68	9	9
SVGF	Hepatocyte growth factor activator (*O. hannah*)	3	V8NIY2	2	2
	Macrophage colony-stimulating factor 1 (*O. hannah*)		V8NW35	3	3
	Tumor necrosis factor receptor (*O. hannah*)		V8P0T5	5	5
SVMP	Asrin (*A. superbus*)	33	A6XJS7	11	6
	BATXSVMPII2 (*B. atrox*)		A0A1L8D6A9	4	2
	BATXSVMPII1 (*B. atrox*)		A0A1L8D600	4	2
	BATXSVMPII3 (*B. atrox*)		A0A1L8D5Z6	4	2
	BATXSVMPIII9 (*B. atrox*)		A0A1L8D641	4	2
	Metalloproteinase (*A. andersonii*)		Q9PT47	6	2
	Metalloproteinase (*D. vestigiata*)		B5G6F7	6	2
	Metalloprotease BOJUMET III (*B. jararacussu*)		Q7T1T3	4	2
	Metalloproteinase PIII (*T. gracilis*)		V5IWF4	2	2
	Metalloproteinase type III 2 (*Hypsiglena* sp.)		A0A098M156	6	2
	Metalloproteinase type III 14b (*Hypsiglena* sp.)		A0A098M219	7	4
	Metalloproteinase type III 14c (*Hypsiglena* sp.)		A0A098M223	5	2
	Metalloproteinase type III 1 (*M. fulvius*)		U3EPC7	5	5
	Metalloproteinase type III 2 (*M. tener*)		A0A194AS47	16	4
	Metalloproteinase type III 2b (*M. tener*)		A0A0F7YYV1	5	2
	Scutellatease-1 (*O. scutellatus*)		B5KFV5	13	7
	SVMP-disintegrin-like mocarhagin (*N. mossambica*)		Q10749	9	5
	Snake venom metalloproteinase kistomin (*C. rhodostoma*)		P0CB14	4	4
	Snake venom metalloprotease (*P.olfersii*)		C6JUN3	4	2
	Snake venom metalloprotease (*P. olfersii*)		C6JUN4	4	2
	Stephensease-1 (*H. stephensii*)		B5KFV4	8	2
	SVMP-Aca-4 (*A. wellsi*)		R4G2D3	11	4
	SVMP-Den-9 (*D. devisi*)		R4FIC4	15	2
	SVMP-Ech-32 (*E. curta*)		R4FJM6	6	2
	SVMP-Fur-1 (*F. ornata*)		R4G2G3	4	2
	SVMP-Hem-2 (*H. signata*)		R4G2W9	8	3
	SVMP-Hop-14 (*H. bungaroides*)		R4FIM1	5	3
	Zinc metalloproteinase-disintegrin-like atrase-A (*N. atra*)		D5LMJ3	14	3
	Zinc metalloproteinase-disintegrin-like atrase-B (*N. atra*)		D6PXE8	9	3
	Zinc metalloproteinase-disintegrin-like cobrin (*N. kaouthia*)		Q9PVK7	7	2
	Zinc metalloproteinase-disintegrin-like kaouthiagin-like (*N. atra*)		D3TTC1	9	3
	Zinc metalloproteinase-disintegrin-like MTP4 (*D. coronoides*)		F8RKW1	11	3
	Zinc metalloproteinase-disintegrin-like ohanin (*O. hannah*)		A3R0T9	19	10
SVSP	Alpha and beta fibrinogenase OhS1 (*O. hannah*)	2	A8QL56	6	4
	Neuroendocrine convertase 1 (*O. hannah*)		V8P1Y2	3	3
	**TOTAL PROTEINS**	150			

Abbreviations: 3FTx: Three-finger toxin; AChE: Acetylcholinesterase; CC3H: Complement C3 homolog; CRiSP: Cysteine-rich secretory protein; CTL: C-type lectin; FMO: Flavin monoamine oxidase; NGF: Nerve growth factor; PDE: Phosphodiesterase; PDGF/VEGF: Platelet-derived growth factor/vascular endothelial growth factor; PLA_2_: Phospholipase A_2_; PLB: Phospholipase B; SVGF: Snake venom growth factor; SVMP: Snake venom metalloproteinase; SVSP: Snake venom serine protease.

**Table 5:** Relative abundance of snake venom toxin families identified in Malaysian *C. rhodostoma* and *O. hannah* venoms. The relative abundance is presented in percentages (%) alongside the number of protein subtypes included in parentheses. Major species-specific families are highlighted in bold (CR, **blue**; OH, **red**).

Snake Venom Protein Families	*Calloselasma rhodostoma*	*Ophiophagus hannah*
3FTx	ND	**31 (55)**
Complement C3 Homolog	ND	**4 (7)**
Kunitz-type	ND	**3 (6)**
Insulin	ND	**3 (5)**
SVGF	ND	2 (3)
AChE	ND	1 (2)
Ohanin/Vespryn	ND	1 (2)
Neprilysin	ND	1 (1)
PDGF/VEGF	ND	1 (1)
SVMP	**20 (23)**	**25 (44)**
PDE	**16 (18)**	**6 (11)**
SVSP	**12 (14)**	1 (2)
CTL/snaclec	**12 (14)**	1 (2)
PLA_2_	**10 (11)**	**5 (9)**
FMO	**7 (8)**	2 (3)
PLB	**6 (7)**	1 (1)
5′-NTD	**5 (6)**	2 (3)
NGF	**4 (4)**	1 (2)
Endonuclease	**3 (3)**	1 (2)
CRiSP	1 (1)	**9 (15)**
Aminopeptidase	2 (2)	ND
QPCT	1 (1)	ND
Trypsinogen	1 (1)	ND
Others (ANK)	1 (1)	ND
**TOTAL**	100 (114)	100 (176)

Abbreviations: 3FTx: Three-finger toxin; 5′-NTD: 5′-nucleotidase; AChE: Acetylcholinesterase; ANK: Ankyrin repeat proteins; CTL: C-type lectin; CRiSP: Cysteine-rich secretory protein; FMO: Flavin monoamine oxidase; NGF: Nerve growth factor; PDE: Phosphodiesterase; PDGF/VEGF: Platelet-derived growth factor/vascular endothelial growth factor; PLA_2_: Phospholipase A_2_; PLB: Phospholipase B; QPCT: Glutaminyl-peptide cyclotransferase; SVGF: Snake venom growth factor; SVMP: Snake venom metalloproteinase; SVSP: Snake venom serine protease; ND: not determined.

## Supplementary Materials

The following are available online at http://www.mdpi.com/2072-6651/10/11/434/s1, Table S1: Complete mass spectrophotometry data of Malaysian *Calloselasma rhodostoma* (Malayan pit viper) crude venom via shotgun-ESI-LC-MS/MS analysis, Table S2: Complete mass spectrophotometry data of Malaysian *Ophiophagus hannah* (King cobra) crude venom via shotgun-ESI-LC-MS/MS analysis, Table S3: Complete mass spectrophotometry data of Malaysian *Calloselasma rhodostoma* (Malayan pit viper) crude venom via GF-ESI-LC-MS/MS analysis, Table S4: Complete mass spectrophotometry data of Malaysian *Ophiophagus hannah* (King cobra) crude venom via GF-ESI-LC-MS/MS analysis.
